# Return to work of transgender people: A systematic review through the blender of occupational health

**DOI:** 10.1371/journal.pone.0259206

**Published:** 2021-11-01

**Authors:** Joy Van de Cauter, Hanna Van Schoorisse, Dominique Van de Velde, Joz Motmans, Lutgart Braeckman

**Affiliations:** 1 Department of Public Health and Primary Care, Faculty of Medicine and Health Sciences, Ghent University, Ghent, Belgium; 2 Department of Rehabilitation Sciences, Faculty of Medicine and Health Sciences, Ghent University, Ghent, Belgium; 3 Department of Languages and Cultures, Faculty of Arts and Philosophy, Ghent University, Ghent, Belgium; 4 Transgender Infopunt, Ghent University Hospital, Ghent, Belgium; St John’s University, UNITED KINGDOM

## Abstract

**Background and objectives:**

Return to work (RTW) or work resumption after a work absence due to psychosocial or medical reasons benefits the well-being of a person, including transgender people, and is nowadays a major research domain. The objective is to examine, through an occupational lens, the literature reporting objective RTW outcomes and experiences in transgender people to (a) synthesize what is known about return to work (full-time, part-time, or self-employed) and (b) describe which gaps persist.

**Methods & sample:**

Several databases and the gray literature were explored systematically. Studies between November 1, 2006 and March 1, 2021 revealing RTW quantitative and qualitative data of adult transgender people were eligible. This review was registered on PROSPERO (CRD42019128395) on April 30, 2019.

**Results:**

Among the 14,592 articles initially identified, 97 fulfilled the inclusion criteria which resulted in 20 being analyzed. Objective RTW outcomes, such as number of RTW attempts, time to RTW or number of sick days, were lacking; thus, other relevant work outcomes were reported. Compared to the general population, lower employment rates and more economic distress were observed, with trans women in particular saying that their work situation had deteriorated. Research on positive RTW experiences was highlighted by the importance of disclosure, the support from especially managers and coworkers who acted as mediators, personal coping, and a transition plan along with work accommodations. Negative work experiences, such as demotion, lay-offs, and discrimination were often prominent together with a lack of knowledge of trans issues among all stakeholders, including occupational health professionals.

**Conclusion & recommendations:**

Few studies have explored employment characteristics and experiences of transgender people (TP). RTW is a dynamic process along with transition in itself, which should be tailored through supportive policies, education, a transition plan and work accommodations with the help of external experts. Future studies should include more occupational information and report RTW outcomes to enhance our knowledge about the guidance of TP and to make way for interventional studies.

## Background

### (Return to) work and health

Work can be considered as one of the determinants of self-worth and a means of social participation and fulfillment. There is a strong association between worklessness and poor health in terms of higher morbidity and mortality [1]. During the last decade, a lot of attention has been given to “return to work” (RTW) research within several psychological and medical settings. An RTW-process is considered a process in which, through assistance and interventions, the worker resumes work after a period of (sickness) absence [2–8]. Ideally, the worker will return to their previous job, with accommodations if required. However, at times it may be necessary to proactively explore alternate or suitable new work during the convalescence period of the worker [2–8].

Emic approaches of RTW research [3–5, 9–11] have already shown a beneficial effect of RTW or work resumption on several levels. On the micro level, returning to work promotes quality of life, community integration and participation of the worker. On the meso level, the company benefits by reducing the costs of recruitment, training, productivity loss, and absenteeism. And on the macro level, society has less expenditure on health care and unemployment costs. There is evidence that vulnerable groups have the same desires as other people and want to participate in society by means of having a job [3–5, 9–11].

### Transgender people and gender-affirming care

Transgender people or persons—an umbrella term for people whose gender identity (GI) or gender expression differs from the sex they were assigned at birth [12]—can be considered vulnerable as they often face difficulties in accessing employment [13–15] and sustainable work [13, 16, 17]. Within gender-affirming care (GAC), some trans individuals choose gender-affirming medical interventions (GAMI) to align their body with their gender [18]. Surgical aspects of GAMI or gender-affirming surgery (GAS) are inevitably accompanied by (several) temporary absences at work, followed by different RTW processes. Even for those not choosing GAMI, the social coming-out process might result in a temporary absence from work. It is therefore not surprising that the “going-back-to-work” process of trans people during social and/or medical transition is met with multiple challenges and the need for support.

### The biopsychosocial model and identity-based model

Occupational health professionals have, among others, the task to assist in the return to employment of those who have been absent. As suggested by the International Classification of Functioning (ICF) biopsychosocial model [19–22], not only biological factors must be taken into account in the dynamic RTW process but also psychological and social factors. For trans people, it is important to consider the overarching theme of gender. Gender is often understood as a socially determined construct and encompasses the set of roles, expectations, and norms that we ascribe to different sexes. In the identity-based model [23], gender variance is understood as inherent to human diversity but in our daily society, trans and gender diverse persons still experience social stigma and less legal protection with associated negative health consequences [24].

### Work experiences of transgender people

Within the transgender health literature, a small body of evidence has focused on negative and positive workplace experiences. Negative experiences (discrimination, micro-aggressions, lack of social support, structural inequalities, etc.) are associated with poor work outcomes and diminished well-being through maladaptive coping mechanisms [24–30]. Yet, a variety of effective strategies (coping methods with strategies, such as identity-based, cognitive, interpersonal and advocacy-related ones) can be utilized to facilitate transitions at work or buffer the effects of discrimination as reported by transgender participants in some qualitative studies [30–33]. Furthermore, other qualitative and quantitative research [26, 27, 34, 35] clearly demonstrates that positive experiences in a supportive and inclusive work climate go hand in hand with higher levels with respect to job performance, satisfaction, and general well-being.

### Objectives and aims

This study is part of a larger project in which it is the goal to optimize RTW counselling for trans people during transition. As the basis for this research setup, our objective was to investigate the international literature on RTW (experiences) by way of a systematic review, and this will be followed by a mixed-methods design with quantitative data and qualitative data of RTW experiences. We searched the literature, using an occupational lens, for the availability of objective RTW outcomes, such as employment status and type, number of work resumptions, as well as (return to) workplace experiences and support during socio-medical transition of transgender employees. This review explores the intersectionality of RTW, transgender people, and transitioning at work (TAW) and may therefore also be useful to (occupational) health professionals seeking guidance on key issues noted among transgender people as well as on assistance in the RTW process.

## Methods

This systematic review follows the Preferred Reporting Items for Systematic Reviews and Meta-Analyses (PRISMA) guidelines [36], for which the checklist can be found in “S1 Checklist.” The methods for the analysis and inclusion criteria were specified in advance and registered in PROSPERO (CRD42019128395) on April 30, 2019. This study protocol can be found in “S1 Protocol.”

Since terminology has changed over the years and is often misunderstood, we have adhered to the current GLAAD terminology [12], where possible, throughout this review, including the reporting of results of included studies. Considering our topic of “return to work,” the term trans(gender) in our review is used as an umbrella term for persons who have chosen steps in transgender health care accompanied by work absence(s) and identify as (trans) women/men, persons with a transgender history, nonbinary persons, genderqueer, gender fluid, agender, and polygender.

### Search strategy

The search strategy was based on the PICOT (population, intervention, comparison, outcome and time) framework [37, 38]. However, due to the absence of a specific comparator and the obsolete timeframe of during or after transition, the search strategy was derived in terms of the participant, intervention, and outcome (PIO). The complete search string was constructed of the combination of four search groups: transgender, gender dysphoria, gender confirmation surgery, and RTW. For each search group, frequently used (old) synonyms, related terms, and database-specific vocabulary were applied. The PubMed database and syntax were used as a starting point for construction and validation. A single database-specific search string example can be found in “S1 Text,” and search concepts along with specific database terms can be found in Table 1. The search strategy was evaluated by the standards set by the PRESS 2015 Guideline statement [39].

**Table 1:** Search terms.

Search group	Thesaurus terms of databases	Keywords or free text words or synonyms	
**Transgender**	Transgender persons (2011–2016)	Trans-gender(s) Trans-gender* Trans-sex* Transex* Transgender individual(s) Transsexual(s) Trans-sexual Trans people Trans person(s) Transgender person(s) Transgendered people Transgendered person(s) Transsexual Transsexuals Transgenderism Trans-gender person(s) Transsex* Gender-variant* Gendervariant person* Gender-variant person*	Genderqueer* Genderqueer person* Transgender person* Trans-gender person* Male-to-female* Female-to-male* Non-binary Nonbinary GNB^a^ Genderqueer Two-spirit person(s) Two spirit person(s) Transsexual person LGBTQ-person(s) Male-to-female Female-to-male FTM^b^ MTF^c^ Trans women Trans woman Trans man Trans men Gender diverse Gender fluid Gender non-conform
	Transsexualism (1968–2012)		
	Sexual and gender minorities (introduced 2018)		
	Transsexualism (added in 1974)		
	Transgenderism (2013)		
**Gender dysphoria**	Gender dysphoric	Dysphoria, gender GI disorder Sexual dysphoria	
	Gender incongruence		
	GI^d^ disorder		
**Gender-affirming therapy**	Sex reassignment procedure	Sex reassignment* Cross-sex hormone therapy Gender reassignment* Cross-sex surgery Sex transformation* Gender change procedure(s) Sex change* Sex alteration* Gonadectomy Mastectomy Orchiectomy Cross-sex hormone treatment Gender-affirming hormones Gender affirming care	
	Gender-affirmation Procedures		
	Gender-affirming hormone therapy		
**(Return to) work**	Work	Work, return to Back-to-work Return-to-work Back to work Work, back to Work retention Labour force Labour forces Labor force Labor forces Employment status Occupational status Work resumption Sick-leave* Disability leave(s)	
	Employment		
	Rehabilitation		
	Vocational Rehabilitation		
	Sick leave		

*Root of term.

GNB^a^: gender nonbinary.

FTM^b^: female to male.

MTF^c^: male to female.

GI^d^: gender identity.

For the purpose of this review, online databases, such as Medline, Embase, ProQuest, Scopus, Web of Science, EBSCOhost, CINAHL, and Epistemonikos, were systematically explored from January 2019 until March 1, 2021 for publications in the fields of medicine, psychology, and sociology. Additionally, other meaningful and gray literature interfaces (i.e., Zotor, OTseeker, and Open Grey) were thoroughly examined within the same time period. References to key articles were manually searched, and their eligibility was examined. Search results were exported in Endnote X8.2 for each database and subsequent deduplication was performed. Two reviewers (JVdC and HVS) screened the titles and abstracts using Rayyan software [40] and performed a full text evaluation. At all stages, in the case of disagreement, a third reviewer (LB) was consulted. There was no blinding for the journal title, study author, or institutions.

### Eligibility

To ensure a comprehensive overview of the topic of RTW in an adult transgender population, all study designs (qualitative, quantitative, and mixed method) with primary or secondary data concerning transgender adults and RTW in English, Dutch, French, and German, but only originating from Europe and Anglo-Saxon countries (USA, UK, Australia), were considered eligible.

The researchers omitted children, teens, adolescents, informal workers, sex workers, HIV-related focus, intersex persons, and persons with dual-role transvestism (2021 ICD-10-CM Diagnosis Code F64.1 [41]) from this review. Informal and sex workers were excluded due to the study’s focus on legal and protected work environments and the regulated process of RTW in occupational (health) care and the emphasis on specific RTW outcomes. Intersex people and persons with dual-role transvestism, who are not defined within the transgender spectrum [12], were also omitted from this review.

Interventions within GAC, such as therapy, procedures, and surgeries, were eligible. No exclusion criteria were implemented in terms of, e.g., type, timing, frequency, or dosage of the intervention. Articles with outcomes not associated with transgender adults and (return to) work (experience) were excluded.

RTW was defined as any work resumption on the part of full-time and part-time, employees, laborers, or self-employed individuals at the previous workplace (same or different job) or in another company or organization (same or different sector). RTW could be eligible as a dichotomous variable (yes or no), a rate or proportion, number of RTW attempts, or time to RTW or number of sick days. No limitations were set on the follow-up period in which RTW can occur in the selected studies nor was a specific assessment tool for RTW upheld as an inclusion criterion. RTW experiences of TP, especially those involving facilitators and barriers to RTW, were established based on qualitative data.

The researchers only performed a screening and full-text evaluation of publications released between November 1, 2006 and March 1, 2021. The year 2006 was chosen as a starting date due to the meeting held in that year, which resulted in the Yogyakarta Principles [42]. These principles triggered developments in the political and social landscape, such as the European Gender “Recast” Directive (2006/54/EC) [43] for the application of human rights in terms of sexuality and GI. It is thus reasonable to assume that this publication and the following directive affected the (return to) work experiences of transgender employees.

### Data extraction & data synthesis

A synthesis of the included articles was performed independently and entailed the following characteristics: author, place and year it was published, the topic, methods used in data collection, participants, most important or work-related outcomes, and work-related key findings.

### Thematic analysis

Qualitative data from qualitative and mixed-methods studies were each separately analyzed by two independent reviewers (JVdC, HVS) in their own narrative. General themes or domains were identified, and testimonies and topics were summarized into subthemes. Similarities in overarching themes and discrepancies were discussed, whereas a third reviewer (LB) was consulted when consensus could not be reached.

### Quality appraisal

Two reviewers (JVdC, HVS) independently performed the quality assessment. The QualSyst tool [44] was used for the assessment of the overall quality of quantitative and qualitative studies with a liberal cut-off for the summary score of 0.55. The Mixed Methods Appraisal Tool or MMAT [45] was applied for mixed-method studies. Disagreements were resolved by consulting a third reviewer (LB).

## Results

### Selection of relevant studies

**Fig 1:**
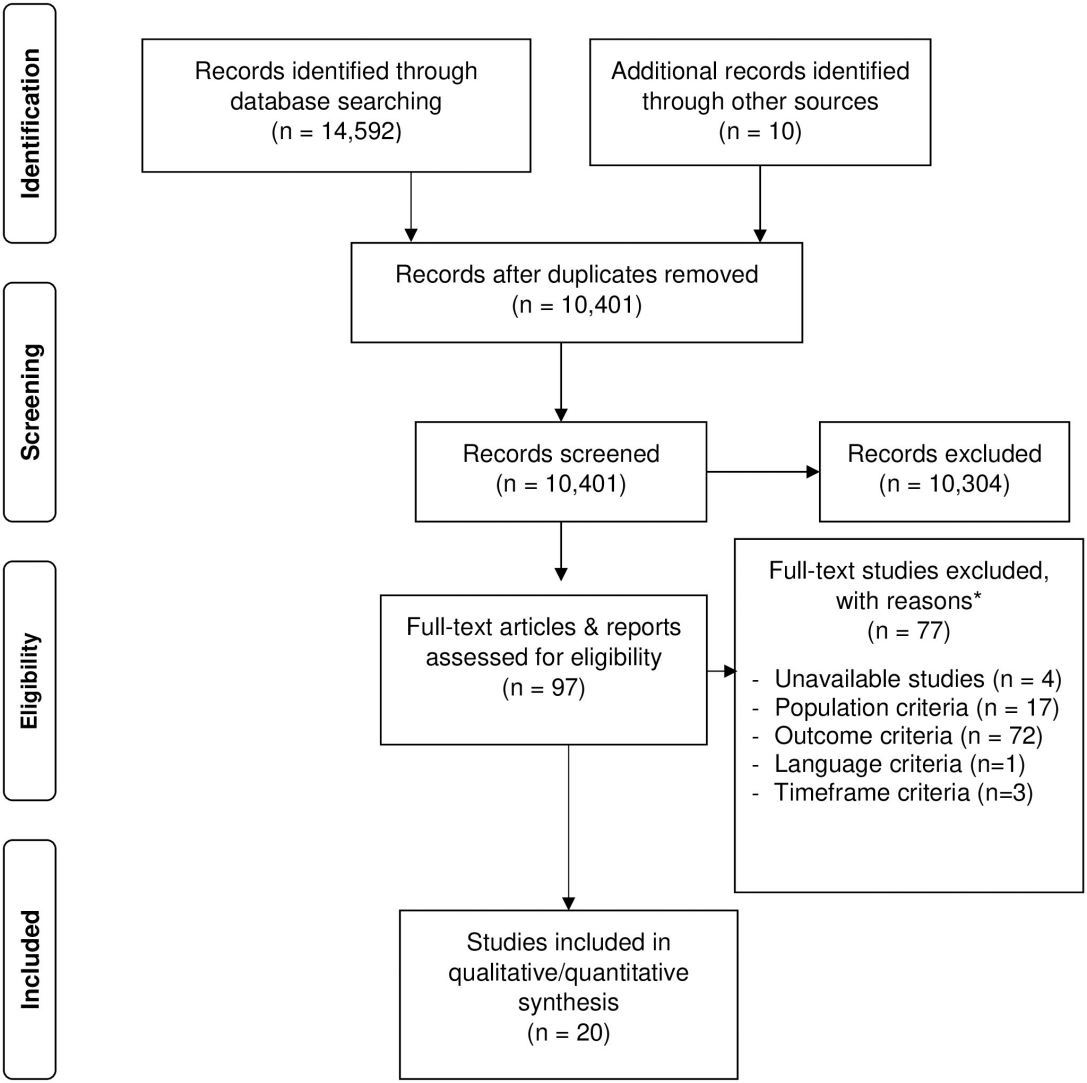
PRISMA flow diagram. An overview of the literature search and study selection [36] *reasons for exclusion: some studies were excluded for multiple reasons.

The flowchart (“Fig 1”), based on PRISMA reporting guidelines [36], depicts the study identification and selection process. The literature research identified 14,592 articles, and ten additional articles were manually searched; following deduplication 10,401 articles remained. For 97 remaining articles, a full-text evaluation was performed and 77 of those were excluded. The articles (77) that were excluded during the full-text evaluation and their reason of exclusion can be found in “S1 Table”. Twenty studies were included for further research.

### Study characteristics and data synthesis

Detailed characteristics of the 20 included studies are listed in the data extraction/evidence table (“Table 2”). The study design included quantitative studies (n = 8), qualitative studies (n = 7), and mixed-methods studies (n = 5). The majority (n = 9) of the studies were conducted in Europe (Belgium, France, Denmark, Germany, Italy, Sweden, the Netherlands) and the USA (n = 6); the remaining studies were conducted in the UK (n = 2) and Australia (n = 2) or were international (n = 1). Eighteen studies were written in English, one study was written in both English and French, and one report was drawn up in Dutch.

**Table 2:** Study characteristics and data synthesis of (return to) work-related outcomes.

Authors, year Country (city)	Methods	Participants	Outcomes	(Return to) work-related key findings
**De Cuypere et al**. [46], **2006 Belgium (Ghent)**	Mixed-methods research Follow-up study 1986–2001 Questionnaires Clinical files Semi-structured interviews Convenience sampling Recruiting TP^a^ post GAS^b^ at transgender health center University Hospital	N = 62 (35 TW^c^ and 27 TM^d^) of which 56 (33 TW and 23 TM) participated in all tests. *x*^e^ age TM M = 33.3 (SD^f^ = 6.8) *x* age TW M = 41.4 (SD = 9.1)	Degree of gender dysphoria (GD^g^) after GAS General functioning Overall psychopathology Treatment outcome Experiences during and after GAS Treatment evaluation	Employment rates before GAS vs. after GAS: TM: sign. (p^h^ = 0.012) more after GAS; TW: no sign. change Unemployment: 14.8% TM and 17.1% TW (= above rate of the general Belgium population) Dismissal: 16.1% in total (20% TW, 11% TM) A larger proportion of patients can benefit from psychotherapeutic support after GAS and should be encouraged to do so
**Schilt et al**. [28], **2008 USA (Austin -Texas, Seattle—Washington, LA -California)**	Quantitative research Three-period panel study (before after recent survey) 2004–2005 Purposeful and convenience sampling (3 trans conferences, online, website advertisement)	N = 43 (27 TM, 16 TW) employed trans adults with positive earnings before and after transition & at current job Control sample: general population March 2003 of the Current Population Survey (CPS) *x* age TM = 30.0, (SD = 2.18) and TW *x* = 39.6 (SD = 2.57).	Working hours Occupation Industry and earnings Experiences (comment section survey)	Profile TP (before transition) comparable to general population but TP more educated Employment types (private, government and self-employment) and pretransition for TW are similar to those of men. More TM in public sector (n = 25, 32%) TW transition ± 10 yrs^k^ later than TM (TW preserving status of men vs. TM attaining status of men) Transition at same workplace = delay of 7 additional yrs Earnings after GAS: TW significant 31% decrease TM a slight 10% increase TP who switched to private sector lost 25.8% Male’s workplace benefits cannot be carried over in transition Workplace gender penalty often accompanies TW’s transition Gender gap in workplace outcomes: not only due to omitted variables; also changed appearance (rather than their changed gender)
**Imbimbo et al**. [47], **2009 Italy (Naples)**	Mixed-method research Cross-sectional Jan. 1992–Sept. 2006 Convenience sampling of TW post-GAS at the clinic Survey at follow-up visit or by telephone (12–18 months post GAS)	N = 139 (TW post GAS or in final step) Adults, *x* age = 31 yrs (SD 5.08), age range: 21–59 yrs	Patient Satisfaction Questionnaire (38Q): general data employment status social and cultural aspects	Employment status: 63% (n = 88) employed, 16% (n = 22) students, and 21% (n = 29) looking for employment. Similar to and a bit higher than earlier EU studies (1993–2008) High work colleague acceptance rates probably due to role of cultural factors or nature of employment Majority of TG patients are not marginalized individuals but active participants, who feel no need to hide their condition
**Budge et al**. [48], **2010 USA (2 Midwestern cities)**	Qualitative research Semi-structured interviews by audio Convenience sampling (Recruitment via e-mail sent to support groups and LGBT university/community centers)	N = 18 (2 TM, 13 TW, 2 female queer, 1 male cross-dresser) *x* age M = 45.17 (SD = 11.51), age range: 20–67 yrs N = 14 white, N = 4 white and native American Current occupations: sales installation farming professional service	Workplace experience before, during, and after transitioning Current job Ideal job Job attainability Ideal career aspirations	Two separate thought processes by participants: Negotiating the course of a TAW^l^ Making career decisions Three transition phases (pre, during, and post transition): each phase held salient experiences related to deciding how to present themselves, physical characteristics they liked or not, and how able they were to present themselves as their new identity and fit in or not be visibly transgender Majority anticipated difficulty after disclosing to superiors, coworkers, and clients. Most were treated better than expected Half described experiences such as being fired, physically threatened, emotionally abused at work due to GI^i^
**Connell C**. [49], **2010 USA, (Austin, Texas)**	Qualitative research In-depth interviews Field journal 2005–2006 Snowball sampling (TG^k^ advocacy group, personal networks of participants, university)	N = 19 (5 TM, 11 TW, 3 genderqueer (GQ^j^) N = 5 not self-identified or visibly TG^k^ Age range: 20–64 yrs white, middle class Employment: I.T. office work blue collar white collar public sector services transport	Work history from first to current job Transition process at work Social interactions of supervisors, coworkers, and clients	Learning to do gender in an “appropriate” way at work: newfound accountability to stringent appearance expectations placed on TP at work No CO^n^ or not visibly TG^k^ < missing legal and social protection The process of transition can bring TP more into alignment with binary gender norms, which can ease others’ anxiety Physical appearance inhibits gender possibility Gender policing Politicization of TG^k^: ‘trans’parency strategy to critique legal and social inequalities Employers evaluate their TG^k^ employees’ performances and abilities in very binary gendered ways Not all TP attempt to undo or redo gender, but all faced the complex task of negotiating the discordance between their sex assigned at birth, gender and sex category (social designation as either “male” or “female” in everyday interactions).
**Johansson et al**. [50], **2010 Sweden, (North, South)**	Mixed-methods research (quantitative setup = prospective longitudinal study) Follow-up (2 years post-op or 5 years in Swedish process): Semi-structured interview + survey 2 trans health centers	N = 42 (17 TM, 25 TW) Diagnosed with ICD-11 HA60 (gender incongruence or GIn) or gender dysphoria (GD, DSM-V) And approved for GAS (> 5 years) +/- completed GAS (> 2 yrs)) Response rate = 70% Age TM vs. TW: at index 28 vs. 37 yrs at GAS: 39 vs. 46 yrs at follow-up 31 vs. 38 yrs TW: late-onset GIn dominated (56%) compared to 11.8% among TM (p = 0.003).	Clinician-reported outcomes: SES work/study status social use of psychiatric care global assessment functioning (GAF) Patient-reported outcomes: SES health social gender role gas experience global outcome evaluation	Employment: 62% TP employed or studying, compared to 21 at the index. The other 16 patients: 2 were unemployed, 2 were retired, and 12 lived on disability pensions (compared to 9 on disability pension at the index) TM were sign. (p < 0.05) younger at first assessment, follow-up, and at time for GAS. Standard rating format (clinicians and patients) Work/studies: N = 15 (36%) improved, N = 21 (50%) unchanged Patients’ statements regarding work situation: N = 17 (44.7%) better, N = 18 (47.4%) unchanged, N = 3 (7.9%) worsened.→ Discrepancy: different evaluation standards More TM were impaired in socioeconomic status (SES) and work, but rated themselves equally satisfied with the outcome as the TW TM already functioning well, and TW had more to gain in terms of social and psychological functioning during the GAS process One TW had a typically masculine job and experienced difficulties coping at work as a woman.
**Parola et al**. [51], **2010 France**	Mixed-methods research Semi-structured interviews Questionnaires Sampling method not mentioned—probably convenience sampling Time period unknown	N = 30 (15 TM, 15 TW) (post GAS (> 2yrs) From different sociocultural backgrounds) Age range: 32–65 yrs	SF36: physical activity perceived health social work mental health personality The Eysenck Personality Inventory (EPI)	Work situation: improved: n = 8 (5 TM, 3 TW); stayed the same: n = 15 (8 TM,7 TW); deteriorated: n = 7 (2 TM, 5 TW) TM have a better social and professional life, better friendships, and better psychological well-being than TW GAS has more positive consequences for TM than for TW: TM have less difficulty in finding their place in society than TW. TM are more stable, adapt better to hormonal treatment, present fewer psychopathological disturbances, fewer surgical complications
**Vennix** [16], **2010 The Netherlands & Belgium (Flanders)**	Mixed-methods study Survey (not validated): Majority of open-ended questions Presumably convenience sampling Recruitment through internet and community support groups and by mail	N = 610 (86 TM, 522 TW) 386 (63% completed the entire survey) Choices of gender-affirming care (GAC^o^): 90 no GAS (28% TM, 62% TW) 54 pre-GAS (34% TM, 20% TW) 56 post-GAS (38% TM, 18% TW) Job sector: policy and administration construction finance and insurance health and welfare trade industry ICT education and training transport TM are on average sign. (p < .001) younger than TW Employment situation: 75% TW have paid work (post-GAS lower than no GAS) Two thirds of TM are employed (but post-GAS much higher, 85% vs. no GAS) Most working TP are employees instead of self-employed (13%)	Demographic characteristics Organizational characteristics Work experience Mental condition Physical health Workplace experiences	Unemployment: 1/10 TP is looking for work, more common among TW; post-GAS are unemployed more often than pre-ops Majority have perception of negative career implications due to TG^k^ background Work absences: On average a minimum of 1 week sick leave due to TG^k^-related personal/mental health factors (63% TW no GAS, 86% TW pre-GAS,74% TW post-GAS) No difference in % within group for not reporting sick last year (42–43%) Average 1-month duration of absenteeism when GAS (34 days TW pre-GAS, TW post-ops 36 days, TW no GAS 14 days) High variation in experiences with occupational physician during (temporary) work absence: Support Good guidance during work re-entry Discrimination No work adjustments But in general more positive evaluation. Female colleagues have in general more positive attitudes towards TP Coming out (CO^n^): especially no-GAS TP don’t disclose TG^k^ background, some postpone, but in general CO^n^ has positive influence on well-being at work Most TP (75–85% TW, 25% TM) do have negative expectations of colleagues TP perceive diversity management as a great contribution to a positive work experience and smooth relationship with coworkers/supervisors. Transgender issues should be included in diversity management and collective labour agreements
**Law et al**. [34], **2011 USA (Philadelphia)**	Quantitative research Survey Convenience and snowball sampling, outcropping Recruitment at conference, online, in transgender community Unclear timeframe (2010)	N = 88 (27 TM, 61 TW) Age: range 19–65 yrs (M = 41, SD = 12.6) *x* age (46 yrs) TW sign. (p < .001) lower than TM (32 yrs) Majority (81%) Caucasian, 15% were minority group members, and 4% did not indicate ethnicity	Identity centrality Organizational support and commitment CO^n^ Coworkers’ reactions Job satisfaction Job anxiety and turnover intentions	Workplace experience better for TM vs. TW (p < .05) = more favorable coworker reactions for TM Emotional costs of not being themselves > costs of leaving Successful CO^n^ and supportive coworkers → TP more satisfied at work + more committed to the organization (p < .01) Supportive organizations stimulate trans employee’s commitment (p < .001) and disclosure (p < .001) Coworkers’ reactions are a mediator between CO^n^ and satisfaction(p < .001), commitment (p < .01), and anxiety (p < .001)
**Jones J**. [52], **2013 UK (Bristol)**	Qualitative research Case report interview Sampling method unknown	N = 1 trans woman (mature elderly) Current age unknown	Experiences in private Experiences work life	Dress codes, difficulties at work and becoming yourself Lack of informing the workforce Better to leave job: Wrong approach supervisor Wrong communication No dealing with coworkers’ behavior HR & supervisor pay lip service to existing TG^k^ policy For 98% of colleagues, there was no deliberate animosity Lack in the pace of change and persistent attitudes towards TP UK Gender Recognition Act, 2004 did not make a big difference
**Brewster et al**. [26], **2014 USA**	Qualitative research Convenience sampling Sample from larger quantitative study [27] Recruited via online resources with 4 open-ended questions	N = 139 (60 men (43%), 37 women (27%), 4 androgynous (3%), 37 other (27%)) Employed adults: 69% full-time (n = 96) and 31% part-time (n = 33) *x* age = 38.5 (SD = 13.2) Age range: 19–64 yrs Majority (86%) is White/Caucasian 92% transition at work and 58% at current employment	Transitioning experiences at work Difficulties and challenges Helpful factors Pieces of advice	In general more supportive work environment than anticipated But nearly all met some form of hostility at work (especially when lacking legal protection): Immediate termination Destructive to career Management sabotage Openly ridiculed Wrong pronouns Harassment Left out Alienated Large part of individuals expressed a smoother transition at work (TAW^l^) than expected. The majority experienced distress/anxiety/depression, but some participants felt empowered and felt TAW^l^ was beneficial and recommended resilience and flexibility (towards coworkers) as coping method Need of external support systems (e.g., activism) and informal psychoeducation on trans issues for a successful outcome The benefits (medical) and protection (legal, policies) of an organization and their accommodations (restrooms, dressing rooms) were central to the transition experience and preparation For nearly half of participants preparation consisted of: legal knowledge backup plans for career administrative changes physical changes Employers: gender sensitive and legal practices decrease negativity associated with gender transitions Practitioners should be equally aware of negative and positive experiences of TAW^l^
**Heylens et al**. [53], **2014 Belgium, (Ghent)**	Quantitative research Prospective study 2005–2009 Questionnaires Presumably convenience sampling (all patients who applied for GAS) Ghent University Hospital and gender clinic	N = 57 (11 TM, 46 TW)(GD diagnosed patients) Age range unknown Response rate for SCL-90 and psychosocial questionnaires were 82.5% and 73.7%, respectively. Follow-up between first and last assessment: M = 39 months (SD: 12.7)	Psychopathological parameters (SCL-90) Psychosocial parameters: employment social contacts substance abuse suicide attempt	Baseline (N = 54): 66.1% employed, 16.1% unemployed, 17.9% other (student, retirement) Follow-up (N = 42): 59.5% employed, 14.3% unemployed, 26.2% other → “other” employment went up, while general employment status went down (TP quit their former jobs and started studying again) GAS has a positive influence on employment (short follow-up period)
**Simonsen et al**. [54], **2015 Denmark, (Copenhagen)**	Quantitative research Follow-up study 1978–2008 Presumably convenience sampling Sexological Clinic, University Hospital	N = 108 (50 TM, 58 TW) *x* age at 1^st^ referral = 28.7 (SD = 9.4), Age range: 14–53 yrs (TW 30.2 yrs vs. TM 26.9 yrs) Age permission GAS: TW 37.4 yrs vs. TM 32.6 yrs Age at start of gender- affirming hormonal therapy (GAHT): TW 32.0 yrs vs. TM 29.9 yrs Group1: employed or students Group2: participants receiving sickness or unemployment benefits on social welfare or pension	Age at transition Self-initiated hormone treatment Years in school Further education Employment	TW sign. (p < 0.001) older than TM at permission GAS Employment at time of referral: Employed TW 62.1% vs. TM 62% TW 20.7% vs. TM 14% on unemployment or sickness benefits TW 17.3% vs. TM 24% on social welfare/pension Employment at the time when permission for GAS was granted: Employed TW 55.2 vs. TM 54% TW 8.6% vs. TM 22% on unemployment or sickness benefits TW 34.5% vs. TM 22% on social welfare/pension Unknown for TW 1.7% vs. TM 2% TW and TM did not differ in employment status Employment rate (54–62%) Unemployment rate higher than general Danish population: supportive Danish welfare system Unemployment rates increased over time: working ability decreased during treatment (no explanation)
**Jones T**. [55], **2016, Australia, (Victoria, Sydney, Melbourne)**	Mixed-method research Emancipatory approach National online survey with multiple choice and open-ended questions + blog forum 2013 (April–July)	N = 273 (TM) Age range: 16–64 yrs (M = 30.5) Self-Identification: 20% TM transgender 7% transsexual male 15% genderqueer 4% other Diverse backgrounds: 77% European Asian 5% < 5% Aboriginal 58% employed: 34% full-time 22% part-time 2% apprenticeship 15% unemployed (higher than general pop. 9%) 33% student (university, vocational education) 20% mental health issues (anxiety, depression, borderline or bipolar personality)	Demographics: age background employment status Identities (allocated at birth, gender id) Work experience	Post-school study perceived as “safer” to transition than the workforce Majority no CO^n^ at work due to: Concern of losing job or missing job opportunities No escape from stress at work & anxiety Fear of social alienation Second-largest group was in transition but: Work avoidance Safe spaces as education Third-smallest group after transition: Not visibly TM and no CO^n^ at work Some career change Acting to conform to work culture General vulnerable feeling regardless of anti-discrimination workplace protections (Australian Commonwealth Law 2013): Support depending on job sector (creative, care-based industries) Some self-employed or on benefits to create supportive contexts Supportive organizations: Good work environment Support management and supervisors Guidelines for colleagues Leadership makes big difference in the employees’ experiences and the workplace GI culture Workplace equity training which should include: Guidelines TG^k^ issues Role for unions Ongoing consultation with staff member about needs of TG^k^ Being flexible in work arrangements
**Ozturk et al**. [35], **2016 UK, (London)**	Qualitative research Explorative approach In-depth interviews Time frame unknown	N = 14 (5 TM, 6 TW, 3 genderqueer) Age range: 28 to 54 yrs 2 persons not visibly TG^k^, 2 persons are to varying degrees visibly TG^k^ Participants work in a range of job roles: construction IT professional services local government retail education charity and healthcare. Various institutional settings, i.e., private, public and nonprofit sector	CO^n^ at work Timeline Scope Process and nature of participant GI transition in the workplace Sources and types of transphobia	Major deficiencies in organizations’ diversity management Workplace knowledge and training deficits regarding GI diversity issues Work relations under strain during transition, some encouraged to take time off due to lack of support Intrusive supervisors, failing to accommodate, common organizational anxiety Organizations unprepared for multiple needs of TG^k^ employee during transition Organizations need to allow: Extended personal leave/career breaks Amendments to employment contract to limit job responsibilities Job-sharing options or similar flexible work arrangements Absence of this creates stress for TG^k^ workers. TG^k^ employees in nondiverse or inclusive industries change career (with compromise in earnings, privilege, and position) Service sector’s work contexts inhospitable due to presumed negative reactions from management, customers and service users.
**Yavorsky J.E**. [56], **2016 USA (Ohio)**	Qualitative research In-depth interviews June–September 2011	N = 25 white TW, > 6 m employment before and after transition 5 GI undisclosed 20 TP out at work or visibly TG^k^ *x* age transition = 41 yrs *x* pretransition employment experience = 20 yrs *x* post-transition employment experience = 6 yrs	Work experience before and after transition	Turnover (TO^p^): In general 46% change job/function 50% change of sector Undisclosed TW (5) switched companies While open TW kept their job or switched companies Cissexism: 50% of the sample with a majority that openly transitioned at the same workplace reported that coworkers viewed them as less competent Reassigning responsibilities or more feminine-typed work, revoking privileges to perform higher status job Coupling of sex and gender determining work assignments and resulting as double binds for TW Some TW believed customers demoted them in social interactions due to their gender
**Marvell et al**. [57], **2017 UK**	Qualitative research Interviews and case studies Presumably purposive sampling, selected based on: Membership of inclusive employers Inclusion in Stonewall’s Top 100 Employers and Knowledge and experience managing TG employees)	N = 16 10 “good practice” employers: majority senior managers variety of public-private sectors, such as: financial government army education housing research & development technology retail 6 stakeholders which represented trans and LGBTQIA advocacy groups and organizations	Outcomes not clearly described: Employment landscape transworkers UK: legislation policy development workplace disputes Good practices employers: policies implementation CO occupational safety and health administration promoting workplace relationships training	Workplace transitions are highly stressful for TP Key practical problems for managing transitions: Records not properly updated and managed Use of staff photos without consent (uncomfortable with appearance) Expectations regarding dress code Access to toilet facilities TP often feel excluded from “social fabric” of organization Bullying, negative treatment, misinformation, and ignorance are still major issues in the workplace and have serious negative effect on the inclusion, well-being and lives Trans staff should have access to safe spaces Organizational awareness, managerial support, better peer attitudes/support, and organizational policies are necessary to improve workplace climates Managers’ attitude and training are essential for reactions of personnel Policies should amplify trans employees’ voices and have an individualized, flexible, and tailored approach, including disclosure towards whole personnel (administrative) Role models/equality or diversity champions play an important role in the organization The return to work (RTW^q^) must be effectively managed, emphasising the organization’s support! Temporary working adjustments during transition and adjusted support should be provided, where necessary, for health-related needs or well-being. Transition plan = flexible plan between line and/or HR managers and the TG^k^ employee. Agreements about communication, work adjustments, work absences (medical, vacation) and RTW^q^, monitoring or frequent meetings (if desired by TG^k^ employee) After TAW, the TG^k^ employee may no longer want/need support from manager The current UK legislation (Gender Recognition Act 2004, Equality Act 2004) does not cover everyone under the wider transgender umbrella Employers should develop policies beyond the legislation
**Gut et al**. [17], **2018 International (Germany, France, USA, UK, Austria)**	Mixed-method research Survey < qualitative + quantitative items Presumably convenience sampling Online recruited via transgender community forums	N = 166 TP (69% identified as female) 91% white Highly educated > 50% with university degree	Employment Support Adjustments	The risk of TP losing job is 3x higher than for LGB workers (organizations do not want TG^k^ representing them) During transition: 59% widely supported, 56% experienced no discrimination Supportive work environment increases CO and job satisfaction Positive encouragement by: 31% senior colleagues 28% same level coworkers 11% juniors Harassment reported by 44%: mostly in organizations lacking diversity training Work adjustments (adj.): 37% no need of adj. 27% positive experience with adj. 6% allowed telework 8% denied telework 24% stated need of adj. but were not offered Average 5 weeks needed for change in work equipment Transition plan: can help managers, the employee and coworkers with managing a transition More than half (54%) with no transition plan 38% company plan 8% long time to implement transition plan 10% many FU meetings Majority (56%) had no follow-up General recommendations: Training HR-managers-staff Diversity manager and implement mentorship Create supportive and inclusive culture Open culture about GI issues Zero tolerance to discrimination Recommendations when employee discloses GI: Tailored transition plan Regular meetings Reasonable work adjustments Communication internal/external
**Meyer et al**. [58], **2020 Germany, (Frankfurt)**	Quantitative research Medical files of an endocrine outpatient clinic from 2009 to 2017	N = 350 TP Employment status: 41.7% employed 14% unemployed Median age at 1^st^ presentation 22 yrs whole sample Median age TW 25 yrs vs. TM 21 yrs	Bio- and socio-demographic characteristics Aspects of legal gender reassignment	Unemployment rate more than twice as high as in general German population TW and TM did not differ in employment status Reverse ratio TW to TM 1:1.89 Decline of psychosocial burden after hormone therapy onset Complex and expensive procedure of legal gender reassignment in Germany
**Bretherton et al**. [59], **2021 Australia**	Quantitative research Survey Convenience and snowball sampling Recruitment: at 2 conferences online in transgender community September 2017–January 2018	N = 928 (37% reported female, 36% reported male, 27% nonbinary), Employment status: 30% full-time employed 24% part-time employed 19% unemployed Median age (IQ range): 28 yrs (23–39) yrs Highly educated: 47% university degree	Demographic data Access to health care Health burden Access to health resources Priorities for government funding	High rates of self-reported anxiety, depression, discrimination especially in health care settings; access to surgery is a major challenge Barriers to employment, double rate of unemployment despite university qualification Better training for doctors in trans issues as priority for funding

TP^a^:transgender people/persons.

GAS^b^: gender-affirming surgery.

TW^c^: trans women.

TM^d^: trans men.

*x*^e^: mean.

SD^f^: standard deviation.

GD^g^: gender dysphoria.

p^h^: p-value, p < 0.05 = significant.

GI^i^: gender identity.

GQ^j^: gender queer.

yrs^k^: years.

TAW^l^: transition at work.

TG^m^: transgender.

CO^n^: coming out.

GAC^o^: gender affirming care.

TO^p^: turnover.

RTW^q^: return to work.

### Quality of evidence

The QualSyst score ranged from 0.68 to 0.91 (M = 0.71) for quantitative studies [28, 34, 46, 50, 53, 54, 58, 59] (see “Table 3”) with an average summary score of 0.77 (SD = 0.08); overall, a score closer to 1 indicates a better quality. For the qualitative studies [26, 35, 48, 52, 56, 57] (see “Table 4”), the overall summary score (QualSyst tool) ranged from 0.19 to 1.0 (M = 0.85, x = 0.70, SD = 0.27): only two studies, with a very distinct study design (report of HR organization, single interview) were of very low (0.19) [52] to low quality (0.50) [57], while the remainder were of good to very good quality. Because of their information value, these two studies were included.

**Table 3:** Quality analysis of quantitative studies with the QualSyst tool.

	De Cuypere et al. 2006 [46]	Schilt et al. 2008 [28]	Johansson et al. 2009 [50]	Law et al. 2011 [34]	Heylens et al. 2014 [53]	Simonsen et al. 2015 [54]	Meyer et al. 2019 [58]	Bretherton et al. 2021 [59]
**Question / objective**	2	2	2	2	2	2	2	2
**Study design**	2	2	2	2	2	2	1	2
**Subject selection or input variables**	2	2	1	2	1	2	2	2
**Subject description**	1	2	2	1	2	2	2	2
**Random allocation**	N/A^a^	N/A	N/A	N/A	N/A	N/A	N/A	N/A
**Blinding of investigators**	N/A	N/A	N/A	N/A	N/A	N/A	N/A	N/A
**Blinding of subjects**	N/A	N/A	N/A	N/A	N/A	N/A	N/A	N/A
**Outcome/ exposure measure**	2	2	2	2	1	1	1	2
**Sample size**	1	1	1	0	1	1	1	1
**Analytic methods**	2	2	1	2	2	2	2	2
**Estimate of variance**	0	2	0	0	1	1	1	1
**Confounding**	1	1	0	1	0	0	N/A	0
**Results**	2	2	2	2	2	1	2	2
**Conclusions**	2	2	2	2	1	2	2	2
**Total sum**	17	20	15	16	15	16	16	18
**Summary score**	0.77	0.91	0.68	0.73	0.75	0.73	0.8	0.82

N/A^a^: not applicable.

The QualSyst tool [44] for quantitative studies: 14 items were scored depending on the degree to which the specific criteria were met (“yes” = 2, “partial” = 1, “no” = 0). Items not applicable to a particular study design were marked “n/a” and were excluded from the calculation of the summary score. A summary score was calculated for each paper by summing the total score obtained across relevant items and dividing by the total possible score.

**Table 4:** Quality assessment of qualitative studies with the QualSyst tool.

	Budge et al. (2010) [48]	Connell 2010 [49]	Jones 2013 [52]	Brewster et al. (2014) [26]	Ozturk et al. (2016) [35]	Yavorsky 2016 [56]	Marvell et al. (2017) [57]
**Question/ Objective**	2	2	1	2	2	2	2
**Study design**	2	2	0	2	2	2	2
**Context**	2	1	2	2	2	2	1
**Theoretical framework**	2	2	0	2	2	2	1
**Sampling strategy**	2	2	0	1	2	2	1
**Data collection**	2	1	0	1	2	2	1
**Data analysis**	2	1	2	1	2	1	0
**Verification**	2	1	0	2	0	0	0
**Conclusion**	2	2	2	2	2	2	2
**Reflexivity**	2	1	0	2	0	1	0
**Total**	20	15	3	17	16	16	10
**Score**	1.0	0.75	0.19	0.85	0.80	0.80	0.50

The Qualsyst tool [44] for qualitative studies: 10 items were scored depending on the degree to which the specific criteria were met (“yes” = 2, “partial” = 1, “no” = 0). Assigning “n/a” was not permitted for any of the items, and the summary score for each paper was calculated by summing the total score obtained across the ten items and dividing by 20 (the total possible score).

Three [17, 47, 51] out of five mixed method articles assessed through the MMAT, generated a variety of “No” or “Can’t tell” on the statements related to the separate parts of their qualitative and quantitative study design, the integration of data and its output, or possible divergences and did not sufficiently adhere to the quality criteria of each tradition of the methods involved. Two studies [16, 55] were overall of very good quality in their mixed methods design, as summarized in “Table 5”. Based on the guidelines of MMAT [45], none of the mixed-method articles were excluded.

**Table 5:** Quality assessment of mixed method studies with the MMAT.

Study design	MMAT^a^ Methodological quality criteria (version 2018)	Studies				
		Imbibo et al. 2009 [47]	Parola et al. 2010 [51]	Vennix 2010 [16]	T. Jones 2016 [55]	Gut el al. 2018 [17]
**Screening questions**	S1. Are there clear research questions?	Yes	Yes	Yes	Yes	No
	S2. Do the collected data allow to address the research questions?	Yes	Yes	Yes	Yes	Yes
**Qualitative**	1.1. Is the qualitative approach appropriate to answer the research question?	Yes	Yes	Yes	Yes	Can’t tell^b^
	1.2. Are the qualitative data collection methods adequate to address the research question?	Yes	Yes	Yes	Yes	Can’t tell
	1.3. Are the findings adequately derived from the data?	Can’t tell	Can’t tell	Yes	Yes	Can’t tell
	1.4. Is the interpretation of results sufficiently substantiated by data?	Can’t tell	Can’t tell	Yes	Yes	Yes
	1.5. Is there coherence between qualitative data sources, collections, analysis, and interpretation?	Can’t tell	Can’t tell	Yes	Yes	Can’t tell
**Quantitative descriptive**	4.1. Is the sampling strategy relevant to address the research question?	Yes	Yes	Yes	Yes	Yes
	4.2. Is the sample representative of the target population?	Yes	No	No	Yes	Can’t tell
	4.3. Are the measurements appropriate?	Yes	Yes	Yes	Can’t tell	Can’t tell
	4.4 Is the risk of nonresponse bias low?	Yes	Yes	Can’t tell	Yes	Can’t tell
	4.5. Is the statistical analysis appropriate to answer the research question?	Can’t tell	No	Yes	Can’t tell	Can’t tell
**Mixed methods**	5.1. Is there an adequate rationale for using a mixed-methods design to address the research question?	Yes	Yes	Yes	Yes	Can’t tell
	5.2. Are the different components of the study effectively integrated to answer the research question?	Can’t tell	Can’t tell	Yes	Yes	Can’t tell
	5.3. Are the outputs of the integration of qualitative and quantitative components adequately interpreted?	Can’t tell	Can’t tell	Yes	Yes	Yes
	5.3. Are divergences and inconsistencies between quantitative and qualitative results adequately addressed?	Can’t tell	Can’t tell	No	Can’t tell	No
	5.5. Do the different components of the study adhere to the quality criteria of each tradition of the methods involved?	Can’t tell	Can’t tell	Yes	Yes	Can’t tell
**Overall quality**		Moderate	Low-moderate	Good	Good	Low

MMAT^a^: mixed-methods appraisal tool [45] includes the responses “yes” or “no” or “can’t tell.”

Can’t tell^b^: this response category indicates that the paper does not report appropriate information to answer “yes” or “no” or that the paper reports unclear information related to that criterion.

### Quantitative results

#### (Un)employment rates, absenteeism and return to work

Less than half of the selected articles reported on employment status (9/20) [16, 46, 47, 50, 53–55, 58, 59] and employment type (6/20) [16, 26, 35, 47, 55, 59] of transgender people. Data on RTW was even more scarce to nonexistent in the case of RTW rates at several time measurements or as a dichotomous variable during transition. Overall, the proportion of transgender people (TP) at work was lower compared to that of the general population and the proportion on unemployment, social welfare, or retirement was higher. Depending on the study and country, the percentage of employed TP varied from 42% to 75% and for unemployment from 9% to 21%. The other participants were either students, on sickness benefits, or retired. In general, transgender men (TM) had a higher employment rate than transgender women (TW) [16, 26, 46, 47, 50, 55, 58] (see “Table 2” for more details).

Regarding employment type, a wide range of job functions was observed in various institutional settings, e.g., health and welfare, education and training, ICT, industry, policy and administration, finance and insurance, transport, construction, trade [16, 35, 48, 49]. Among pretransition TW, the number who held a job in the private sector, government, or were self-employed was similar to that of all TM [28].

At the time of transition, TW were on average 5 to 10 years older than TM, but TP who stayed at the same workplace waited up to an additional 7 years to transition than those who had changed jobs [16, 28, 34, 46, 50, 58]. In three studies [26, 35, 56], the majority of TP openly transitioned at work and about half at their current job [26]. Less than half of trans employees had a transition plan or were granted work adjustments [17]. On average, TP were absent for a minimum of 2 weeks due to transgender-related psychosocial factors and on sick leave for 1 month after gender affirming surgery (GAS) [16]. The majority of respondents (70%) had a positive experience with their occupational physician (e.g., guidance and support) during their work absences.

After transition, a significant number of transgender persons lost their job (6–27%) [16, 17, 46], were demoted (10–24%) [16, 17], held another employment status, or switched jobs (12–46%) [16, 50, 53, 54, 56]. For some transgender employees, absenteeism formed the official reason for discharge [16]. In half of the cases, a turnover in either job, company, or sector was observed [28, 34, 56].

More TM described their work situation as improved, while TW were more likely to experience a deterioration [50, 51]. The discrepancy in income and reactions from coworkers are illustrations of these different work experiences [28, 34, 51]. Transgender women encountered an average decrease of 31% in their income, while TM gained on average 10% [28]. This factor maintained its significant impact, even when years between the observations, obtaining a college degree since the first observation, changing from a white-collar to a blue-collar job, or changing to a private job were taken into account.

### Qualitative results: A thematic analysis of (return to) work experiences

During the thematic narrative analysis of (return to) work experiences from the qualitative data of twelve studies, two overarching main themes emerged: coming out (CO) at work and transitioning at work (TAW) (see “Table 6”).

**Table 6:** Thematic analysis of (return to) work experiences.

Major themes	Subtheme	Sub-subtheme	Examples	Studies
**Coming out at work**	**Perceptions & expectations**	Supportive vs.^a^ nonsupportive organizations	*Policy* *Benefits*	[17, 26, 35, 48, 55]
		Social &^b^ private coming-out experiences	*Stigma* *Support*	[16, 26, 35, 48, 55]
	**Preparation**	Legislation	*Country* *Region*	[17, 27, 48, 55]
		Communication	*Sit-down HR/supervisor* *Safe spaces*: *Person of confidence* *Counsellor* *Union* *Diversity/equality expert*	[26, 48, 55]
**Transitioning at work**	**Reactions: HR and supervisor**	Knowledge of transgender issues	*Awkward questions* *Forced medical leave* *Diverse & inclusive workplace*	[35, 48]
		Knowledge of policies	*Lip service* *Diversity & equality management* *Training*: *Communication* *Diversity*	[17, 35, 48, 55, 57]
		Support	*Policy beyond legislation* *Education* *Inclusive workplace* *Exemplary attitude managers*	[16, 17, 26, 35, 52, 55, 57]
		Negative career implications	*Dismissal* *Declined promotions* *Perceived need of safeguarding the workplace from trans employees in specific gendered stereotyped situations* *Forced vacation or medical leave* *More oversight* *Salary (TM vs*. *TW)*	[16, 17, 26, 49, 55]
		Binary thinking	*Diminished expectations (job function vs*. *gender)* *Overprotection* *Uniforms* *Agreement regarding toilets & dressing rooms*	[17, 26, 35, 48, 49, 56, 57]
	**Reactions: coworkers**	Harassment, discrimination & stigma	*Name-calling* *Wrong pronouns* *Wrong names* *Social alienation* *Complaints of toilets & dress rooms* *Cissexism*	[16, 17, 26, 35, 48, 49, 55–57]
		Support	*Helpful* *Acceptance*	[16, 17, 48, 55]
		Policing gender	*Stereotyping* *Work culture*	[17, 26, 48, 49, 52, 55, 56]
	**Transition plan**	Administration	*Data* *Privacy* *Social presentation*	[17, 57]
		Communication throughout organization	*Self* *Meeting* *Email* *Sensibilization sessions & presentation*	[17, 57]
		Cooperation	*Transgender employee* *Human resources* *Managers (senior*, *line)* *Union* *Third parties*: *Medical* *Occupational health & safety* *Trans community* *Counsellors* *Legal*	[17, 26, 48, 55]
		Health	*Medical leave/sick days* *Trans health-related factors*	[16, 57]
		Work adjustments	*Hours* *Tasks*	[17, 35, 55, 57]
		Protection and job security	*Antidiscrimination policies*	[26, 48, 55]
	**Coping mechanism**	Taking action Seeking social support	*Seeking support* *Activists* *Legislation* *Equality experts*	[26, 48, 55]
		Avoidance and palliative reactions	*Seeking diversions* *Danger of addiction*	[60]
		Emotional	*Depression & anxiety*: *Brooding* *Self-doubt* *Social isolation* *Suicidal ideation* *Frustration & anger*	[16, 26, 48, 55, 57]
		Overcompensation		[48]
	**Appearance & personality**	Visibly vs. not visibly transgender	*Physical* *Expected stereotyping*	[16, 35, 48, 49, 55–57]
		Changing interests	*Work content* *Education (internal)*	[55]
	**Personal career choices**	Turnover	*Private vs*. *public sector* *Intersectoral* *Job functions*	[16, 17, 35, 48, 55, 56]
		Going the extra mile	*Working more hours* *Being a model employee*	[48]
		Education	*Skills for new job prospects*	[55]
		Compromise and accommodate	*Settling for lesser pay in different organization* *Taking any work you can get*	[16, 55]
		Work absences	*Unpaid/paid leave* *Career break*	[16, 17, 55, 57]
		Unemployment	*Resignation and wait until after transition*	[16, 17, 55, 56]

vs.^a^: versus.

&^b^: and.

#### Coming out at work

The two subthemes which were extracted from the data associated with the period/phase of CO at work included preparation and outcome expectations. Within the subtheme of making preparations, topics related to external resources, legal help, and communication were involved.

### Preparation

Budge et al. (2010) [48] and Brewster et al. (2014) [26] observed that a majority of transgender employees felt the need to prepare themselves for their transition. There were different measures and several decisions to make concerning when and how to disclose and present to others.

#### External resources and legal help

Before TAW, most transgender persons tended to disclose in their private life first [48]. If this personal/social CO was a positive experience, and encouragement and support from friends, family members, etc. were experienced, individuals felt it less difficult to come out at work [26, 48].

Before CO, transgender employees searched through literature or online sources to find out what the transition process had been like for other transgender individuals. In this regard, they learned from the experiences of others and they thought well in advance how to protect themselves from potential negative consequences. Some also sought legal help to find out and understand what their rights were, or consulted antidiscrimination policies in order to make informed decisions [26, 48].

#### Communication

To start disclosure at work, three common communication strategies were identified in the qualitative studies. A first strategy, chosen by only a limited group, was to inform more or less everyone at once [16, 26]. However, for many transgender employees, their first disclosure at work was with someone with whom they felt safe. Therefore, the second and preferred way was to go “top-down” and start discussing the transition with a supervisor, an HR manager, a confidential advisor, or a union representative, who then often helped with the further CO. A third strategy was a kind of “bottom-up approach” by first disclosing to coworkers with whom the transgender employee was most familiar before notifying the HR department [16, 26] (2010).

During these first encounters, transgender employees also discussed their options regarding CO at work fully and the timeline for being able to express their GI through make-up, clothing, etc. [48].

Throughout this preparation stage, contacts with occupational health professionals (occupational physician, company doctor, or vocational psychologist) were only mentioned by Vennix [16]. CO during consultation happened with regard to a “top-down’” communication or work absences.

### Outcome expectations

The lack or presence of organizational awareness and support is a decisive factor for employees with respect to (non)disclosure (of transgender history) and TAW [17, 35]. Several studies have indicated that transgender employees who expected little or no support at the workplace hid their transgender identity and waited as long as possible to come out at work. They anticipated harassment, discrimination or stigmatization from their supervisors, coworkers, and clients due to disclosure [26, 35, 48, 55, 57]. All transgender employees in general feared that they would lose their job, be passed over for promotions, or be demoted [17, 55, 56] which often led them to postpone their disclosure and transition although some TM tended to invest in disclosure because of work seniority [55]. Avoidance of disclosure as well as negative anticipation caused for many symptoms of distress, such as anxiety, depression, and suicidal ideation [48, 52, 55]. A disclosure disconnect between private and work life can also cause extra strains on (psychological) well-being [31].

In contrast, organizational support and acceptance in creating an inclusive work climate made CO easier than expected. Diversity management integrating GI as a significant category, organizational policies and guidance to support and value gender and sexual minorities were essential keys to facilitate disclosure decisions of transgender employees [35, 55].

Transgender persons perceived specific sectors as more supportive, such as the care and creative sectors [55]. Sectors such as industry, construction, and services had a nonsupportive reputation, which inhibited TP in these sectors from coming out at work [35].

#### Transitioning at work

The six themes which were extracted from the qualitative data regarding TAW were reactions of management and HR, reactions of coworkers, the presence or absence of a transition plan, coping mechanisms applied by the transgender employee, changes in appearance and personality, and personal career choices. Each of these themes is described below, but in general both positive and negative aspects of TAW were observed for all topics. However, some studies reported how transgender men had a better professional life and benefited more from transitioning than transgender women through the exchange for the perceived “higher gender” in a binary society [48, 49, 51], whereas research focused solely on TM has mentioned the difficulties in navigating expectations involving masculinities [55].

### Reactions from management and HR

#### Knowledge of transgender issues

The reactions from management and HR played an essential role in the TAW. Five topics were found: knowledge of transgender issues, implementation of policies, support, negative career implications, and binary thinking.

Although managers and HR professionals were the first point of contact when an employee disclosed a gender TAW, some could not recollect having had to support a transgender person in the process and lacked knowledge and understanding of trans issues [17, 57]. For example, they were unaware of the types of accommodations or changes that needed to be made for transgender individuals. Therefore, studies were in consensus that HR (managers) must have up-to-date information about good practices and be trained on how to manage a gender transition in the organization [17, 48, 55, 57]. A positive observation was that if an organization lacked knowledge on transgender issues, they undertook steps to find out how other companies handled the transition process and provided quality training to managers focused on GI and workplace discrimination [48, 57].

#### Implementation of policies

Various studies reported that organizations and managers had indeed taken steps to develop a diverse and inclusive environment. They worked with external experts and by preference with input from trans employees to provide access to information, advice, and training sessions on diversity [17, 26, 52, 57]. However, simply developing such a policy and drawing up gender transition guidelines were not sufficient, as it did not prevent some management and HR departments solely paying lip service and failing to inform the workforce, not deal with inappropriate coworker behavior, or not take into account the employee’s needs and requirements during transition (e.g., privacy concerns) [16, 17, 52]. A time frame of one month up to one year was not unusual for the desired changes in administration (email, name tags, business cards) or work equipment to happen [17].

Therefore, such policies require genuine engagement on the part of the management and HR, and they should be communicated across the workforce to ensure that good practice is consistent and implemented across the organization [17, 57]. In addition, transgender employees emphasized that these policies and approaches must be individualized, flexible, and tailored because there is no such thing as “the transgender experience” [57].

#### Support from supervisors and HR

In general, transgender employees experienced more support and better treatment than expected [16, 17, 26, 48, 55]. Organizations and HR managers were perceived as supportive by their transgender employees when they, for example, created appropriate work adjustments, informed and distributed guidelines among coworkers, organized meetings and provided equality/diversity training for the workforce (e.g., emphasizing the difference between GI and sexual orientation), promoted LGBTQIA+ events and workplace champions [17, 26, 48, 52, 55–57]. Managers and supervisors gave transgender employees a feeling of acceptance by setting an example, which was crucial for the transition experience. Among other ways, they did this by using the correct pronouns and by treating the individual as usual [17, 26, 48, 49, 55].

#### Negative career implications and binary thinking

Regardless of the differences between TW and TM, all employees were at some point inundated with the fear of dismissal. These feelings would be emphasized by previous experiences on a societal or private level, the perception of a transgender unfriendly organization, and experiences of other LGBTQIA+ minorities. Some transgender employees did report being aware of a possible negative impact because of their transgender (TG) status, such as having trouble concentrating at work, being preoccupied with their transition [16]. As their competencies were being questioned literally or they experienced more oversight of their supervisors, TP were no stranger to being passed over for promotions, being demoted or “let go” for a stated reason other than their GI.

As binary thinking and gender hierarchy are still a norm on a societal level/organizational level [49, 56], employers quickly raised questions about job fitness in relation to GI. In more masculine functions, doubts were formulated by supervisors whether the transgender employee could still perform previous job tasks (e.g., computer coding) or if it was still appropriate for the employee to continue their work in certain context (e.g., male attendance in a children’s book section). On the other hand, some employers proved to be overbearing towards physical tasks to be executed by TW. Managers would frequently disagree about the use of restrooms, dressing rooms, or gendered sporting facilities, for example, gender-neutral spaces were an infrastructural hassle and a subject of distress for transgender people.

### Reactions from coworkers

The reception of trans employees by the workforce during their transition period was a recurrent theme. These interpersonal relationship experiences were characterized by three main topics: discrimination and policing gender and also the display of genuine support.

#### Discrimination/harassment/stigma

The majority of transgender employees had to deal with direct or indirect discrimination by coworkers. Most reported acts of misconduct by coworkers were “dead naming”, the use of the wrong pronoun (“misgendering”), and asking inappropriate questions. Some trans employees linked this to a gross lack of knowledge about transgender issues. Frequently, the stigmatization of being transgender led to actual harassment, such as name-calling, being reported by colleagues, being questioned about one’s abilities, being excluded from the social constructs of the organization or getting complaints concerning the “inappropriate” use of restroom and dressing room facilities. Some work branches were more susceptible to being a transgender-unfriendly environment or for mobilizing masculinity, which led some nondisclosed transgender employees to join in this behavior just to belong. In some studies, there was a specific reference to “cissexism,” which was mainly reported by TW. As such, some cis coworkers did not shy away from discriminating against TW for being transgender and being a woman, which was viewed as the “lower gender” in a binary system as for most colleagues the notion of “gender blending” or a nonbinary gender construct was off-putting and beyond their grasp. Three studies [17, 55, 57] emphasized the importance of union representatives for the application of antidiscrimination laws and policies.

#### Policing gender

Transgender employees reported how they received remarks on how to appropriately behave as a man or woman (gender policing). Some TP believed that coworkers took it upon themselves to teach them how to dress properly as an initiation course or “apprenticeship” [49] to the correct gender expression in relation to their GI. These situations where transgender employees were expected to conform to the norm proved to be a great source of distress. TW often felt constrained in their behavior, as it was not rare to be labelled as aggressive or dominant (e.g., being a bitch) when displaying more “masculine” traits or knowledge (e.g., being able to talk about cars). Especially in specific industry sectors with a macho culture, TW could be “punished” for not being one of the guys anymore, while some TM experienced that coworkers would voice that their presence had become abhorrent (e.g., primary education, support services for women). It was not unusual for those organizations to have undercurrents of homophobia and transphobia [26, 49, 55].

#### Support

A proportion of transgender employees in different studies acknowledged the welcoming support of many colleagues. Especially ciswomen were more prone to acceptance and considerate of trans employees’ needs and feelings. Support was often experienced as being respectful by using the chosen names and pronouns, acting like it was business as usual and continued socializing (e.g., coffee breaks, drinks after work).

### Transition plan

When transitioning at the current workplace or when discussing policy implementation and good practices, several articles focused on a transition plan. Within this domain, six elements were recurrently discussed as a necessity (to be included): the regulation of administration, modes of communication throughout the organization, the importance of cooperation between several actors, health-related factors, the agreements and application of work adjustments, protection of the transgender employee, and their job security. Studies emphasized that a unique tailored transition plan was a clear way to help guide managers and their workforce to support the employee [17, 57] as some transgender employees sometimes felt constrained by too much control provided by the plan and insufficient flexibility towards their changing needs throughout their transition [35].

#### Administration

All transgender persons underlined the importance of the administrative changes within the organization as a part of their true self, their social identity within the company, and towards clients. Studies advised a speeding up of this process (email, name tags, data) by not waiting for legal changes implemented by municipalities. Their GI history was often a concern; therefore, privacy protection was deemed by several studies to be an essential routine procedure to be implemented [17, 26, 48, 57].

#### Communication

Agreements concerning communication about gender TAW are to be made and drawn up in the transition plan beforehand. Various methods and combinations can be used for this purpose, but all studies concluded that the decision should be made by the transgender employee. Some chose to disclose this one on one or in the company of the team leader, while others preferred this to happen in an organized personnel meeting sometimes preceded by a diversity training or specific transgender-oriented information session. In other instances, due to practical reasons and the preference of the employee to avoid such a social (and possibly unnerving) situation, disclosure was done via email or a letter. Aside from disclosing the true GI of the employee, communication about chosen pronouns, respectful behavior and sensibilization were also brought up as part of the content of a transition plan.

#### Cooperation, protection, and job security

Studies mentioned the transgender employee as a central figure in the makeup of the transition plan alongside HR and management. Few studies referred to the involvement of the union to guarantee protection against negative career implications, the application of employee’s rights, and antidiscrimination policies. Only two studies [26, 57] mentioned collaboration with third parties to draw up the transition plan. The majority of employers and employees found the legislation of different countries (Europe, US, UK, AUS) insufficient as a backbone for adequate policies, impractical to implement, or not all-inclusive [17, 52, 55, 57]. Experts on transgender rights, advocacy, and support groups can be leaned on for legal guidance and also for the education and support of managers.

A schedule of follow-up meetings through transitioning was deemed important by many employers and some employees, while others preferred less informal ways for a manager to keep up with their transition and needs; a few mentioned never having had any interaction afterwards.

#### Health

The transition plan entailed the handling of work absences due to gender-affirming treatment, whereby discretion and flexibility were emphasized by employers, employees, and advocacy groups. Some transgender employees preferred taking a longer break while transitioning. Marvel et al. [50] specifically stated that the RTW after each absence is to be effectively managed and supported by the organization. Health and well-being related to TAW can be supported in a general (occupational health services, employee assistance programs) or tailored way (third parties with transgender expertise) as reported by employers [57]. Some TP, however, preferred to seek outside (private) help, which can be included in the transition plan and should be treated as a paid work absence as for any health appointment. Transgender persons did emphasize that while some have (mental) health problems due to gender dysphoria, this is not to be generalized. When applicable, health benefits (e.g., a company’s health insurance policy, country legislation) are to be discussed and recorded in the plan. The issue of medical costs weighed a lot on transgender employees’ minds, as some even had to delay some steps in their transition to save up enough money.

Health issues with absenteeism were mostly of a psychosocial nature (e.g., personal, family, sleeping and concentration problems) due to their transgender status. Mental well-being was strongly related to work experiences, relations with coworkers, and the presence of a culture of diversity at work. During these long work absences, managers and colleagues were mostly supportive [16].

#### Work adjustments

The setup for (temporary) adjustments could entail physical arrangements (e.g., use of dressing or restrooms), job mutation (e.g., working in another job capacity or other location), working a different schedule (other work hours or fewer), performing other or less tasks in the same job (e.g., light duties after chest surgery), and different performance standards for evaluations during the transition. Especially in customer- or client-based sectors, some transgender employees did not feel comfortable and preferred working at another location or from home during their transition [57], as in some cases call-based contacts could also lead to distress [56] (e.g., voice and gender association by clients). In one study [16], work adjustments were part of a reintegration plan drawn up by the occupational physician (or company doctor), and the majority of transgender employees felt greatly supported and understood. On the other hand, some hit a brick wall and even felt discriminated against when they were frequently absent (for long periods of time) from work (e.g., absenteeism) [16].

### Coping mechanisms

During their TAW, employees reacted in different ways. These were categorized as the following: taking action and seeking support, avoidance and palliative reactions, emotional coping, and overcompensation mechanisms.

Transgender employees, with a more active coping mechanism, did not refrain from addressing coworkers regarding their awareness of transgender issues or their behavior while they also tried to remain flexible and patient towards their colleagues. Some felt empowered by this and did not have to struggle as much during their TAW. Others focused on the importance of changing a work culture and augmenting diversity and equality. While for some activism seemed natural or a given, organizations should not automatically expect such an investment from a transgender employee. Some trans employees chose to get things off their chest through their social support network, while others sought the help of counsellors, lawyers, union representatives, and the transgender community. This was a dynamic process from before the disclosing of their GI to discussing matters of rights, policies during transition, up to career issues post-transitioning [26, 48, 55].

Positive coping mechanisms were reinforced by positive reactions from the work environment [26], but these actions could also be part of a back-up plan together with the training of new skills and the prospect of a job mutation or turnover [26, 48].

Some transgender employees tried to overcompensate for their transgender status by working overtime or being extra grateful for being employed [48]. Many trans employees reported distress, feeling insecure, having anxiety, or feeling depressed [16, 26, 48, 55], which was predominantly related to negative reactions from their surroundings but not necessarily so [16, 55]. Some described being in a negative spiral that ended in sanctions, demotions, job loss, or even suicidal ideation [47, 48, 53, 55].

### Appearance and personality

From the qualitative data, a great deal of the experience of TAW took place at the level of appearance and personality. Transition meant becoming their true self on the exterior as the interior. For most TP the association of their GI with their visible physical characteristics, as perceived by others, was a source of distress and anxiety, while for others the feeling of “being free” dominated [26, 35, 48]. Nearly all were tied up with their gender expression (e.g., through clothing, hairstyle, make-up, composure) and/or chosen steps in transgender health care (GAC). Generally, as the age of transition was higher for TW and due to male body characteristics, they were frequently confronted with their gender history by reactions from others [26, 48, 49, 52]. For some, this resulted in the beginning in a misconception that femininity should be characterized by external female traits [52]. Other TP chose to undo binary thinking at work through “gender blending” [49]. TM in specific sectors (e.g., working-class culture, transportation, construction) were also challenged in their behavior (e.g., be an “authentic” man). Apart from physique and behavior, many TP also displayed previously hidden interests and personality traits during their transition and afterwards (e.g., engaging in training, new social experiences, job tasks, asking for a posting that better suited their true self) [16, 26, 48, 56].

### Personal career choices

In nearly all studies, trans employees decided their career pathways based on their perceptions, experiences, and personality. These career choices entailed turnover, compromising, and accommodating for less through education, by going the extra mile at work, or being a model employee, being absent from work, or being unemployed.

Many transgender persons choose the way of turnover between jobs (within the same company), organizations, or sectors based on changing interests more in harmony with their true identity, or based on a transgender-friendly environment (e.g., work culture in specific sectors, diversity policies, legislation in specific geographic regions) or to have a clean break from their gender history [16]. Some preferred to extend their skills by following post-graduate education [25, 26] or specific job training programs [16]. Through turnover to jobs in the public sector or extended education, trans employees often settled for less on the level of income; many would even quit their job to be able to transition in a more comfortable way or because of the very gendered work environment they were in. Some would prefer to take a career break or take an extended (un)paid leave during their transition. When organizations were deemed transgender friendly, or when employees felt a sense of loyalty towards their job/employer, or were invested due to work seniority, some would work extra hours, try to be an amiable and supportive colleague, or be extra productive, whereas others would invest in raising awareness through trans activism within the organization and help establish policies.

## Discussion

This study aimed to synthesize—through the blender of occupational health—the relevant literature on work outcomes and RTW (experiences) in the trans community in order to identify gaps relating to the RTW process and the guidance from occupational health professionals. Core findings were the lack of objective RTW data, the need for tailored management and a transition plan, seeking safety in education and planned work absences, and a dominant presence of binary thinking, which was reflected in gendered stereotyping. Although some studies could highlight positive transition experiences at work due to supportive organizations, the advice of the occupational physician in creating such a work environment was rarely asked by management and evaluated by researchers.

### (Return to) work outcomes

Our first research question entailed the procuration of an overview of objective RTW outcomes, such as number of sick days during or after gender affirming care (GAC), time to RTW, and RTW attempts. Unfortunately, apart from a short mention of absenteeism in Vennix [16], such precise data remained elusive. As such, we focussed our quantitative synthesis on more general work-related outcomes, such as employment rates, turnover, age of transition, and objective career-related outcomes following disclosure and TAW.

Employment rates were in general lower, with up to one third working part-time and unemployment being much higher than the general population of each country. This was even more true for trans women (TW) than for trans men (TM) [28, 50, 51], but unemployment rates should also be mirrored in the transition phase and chosen steps in GAC. More transgender people (TP) were also either students, on disability, on welfare pensions, or retired, although the proportion of TW versus TM therein was mixed [16, 54, 58]. Countries with a strong social security system, such as Denmark, Sweden, or Belgium [46, 50, 53, 54], included more TP on social welfare or sickness funds. Parallelwise, this could be attributed to insufficient protective legislation, reported discrimination, and a negative work environment, lack of awareness of the labor market and in organizations, administrative difficulties, and mental health conditions.

Only a minority of studies disclosed the job titles or sectors in which their participants, before and after transition, were active. Nevertheless, our results showed a shift among TM from white-collar (WC) jobs [28] or the health sector [16] and TW in government or public sector jobs [16, 28] a decade ago to a recently more prevalent presence of TW in blue-collar jobs [56]. This could be attributed to a changing work culture, the sociopolitical landscape and more diversity management in otherwise “masculine BC jobs”. Turnover (TO) and TO intentions, which were also underreported, of trans employees in Belgium and the Lower Countries (17%) [16] were more in line with the general population (20% Belgium [61]), while US trans participants had a higher turnover (50% vs. 27% in 2018 for the general population [62]). The heterogeneity of (lacking) legislation, changes in work culture, the sociopolitical landscape, and a more diversity-oriented management as well as trends of involuntary part-time work [63] of TP may largely explain these observations.

Although the number of sick days, periods of long absences, and time to RTW are important factors throughout transgender health care, only one study [16] mentioned exact numbers for an average sickness absence, namely 2 weeks on yearly basis and 1 month after GAS. Psychosocial factors related to the transgender status of participants were herein an important contributor to sickness absence. According to the US Bureau of Labor Statistics, the overall absence rate was circa 3% in 2020 [64], while in Europe, average rates varied between 3% and 7% [65–67]. Higher absence rates in Europe can be explained by higher levels of unionization and social security laws that protect employees. Due to insufficient data, it is difficult to compare absenteeism or even short-term absences of TP with the general population.

Part-time employment (involuntary) along with prolonged education, living on unemployment or welfare benefits, discrimination and turnover to less lucrative jobs, cause TP to be more likely to suffer from economic stress [13, 63]. When mentioned, household income or socioeconomic status [26, 28, 55] of TP was mostly below the annual national average income [68, 69]. In general, trends showed a profitable gaining of income for TM as they “upgrade” to a “higher human capital” or a status quo when already working in good job positions pretransition, while TW lose their “male advantage” and earn one third less [28]. Socioeconomic concerns were a major instigator to delay transition of TW, which is reflected in an average older age of transition (≥ 10years) [16, 26, 28, 34, 46, 50, 55, 56], although two studies [54, 58] reported a smaller age gap of 4–5 years between TW and TM, which could be attributed to the long process of authorization to GAC in their respective countries (Denmark, Germany) at that time, wherefore TP would have been prompted to contact transgender health care services much earlier. As seen in Meyer et al. [58] and Bretherton et al. [59], a general younger trans population was also reached through social media for recruitment or as a way of pre-GAC information.

### (Return to) work experiences

The second main goal of this review was mapping the experiences of trans people, when they (return to) work during and after transition, through a narrative thematic analysis. As with the quantitative data, we could only find two reports [16, 57] mentioning RTW, while in other articles we had to extract work experiences in general of TP, which we, as vocational experts, could indirectly relate to work retention or RTW.

Based on the results of our review, the experiences with the personal disclosure and support system, influences from current activism and legislation gave root to the perceptions, expectations, and motivations of the individual about CO and TAW. The importance of TP’s perceptions or expectations and actions therefore can be framed within stigma theory, already described by Goffman [70] in 1968 but still relevant, and within the division into three types by Link-Phelan [71], such as self-stigma, experienced stigma, and perceived stigma. All three types were found in our thematic analysis, whether it was a perception of negativism and the decision to quit their job or social isolation or taking time off work or being discriminated against by colleagues or managers. Such findings are in line with a recent meta-synthesis of social integration and well-being of TP [72].

Although TP are generally perceived as a vulnerable group and literature has a tendency to highlight negative aspects of CO and transitioning, for some the aspect of no longer living with a secret relieved their distress, empowered them, and boosted their confidence, which in turn influenced their behavior at work and coping. As such, they could break the vicious cycle of stigmatization. Positive thinking has been a focus in (organizational) psychology [73, 74] and is also an important personal factor to facilitate going back to work [75, 76] while motivation (autonomous or controlled by external factors, e.g., financial) plays an important role in the RTW process [77, 78] and should be evaluated, supported, and guided by (mental) health professionals.

Additionally support, which has been long known as a social determinant of health [79], on any level (emotional, instrumental, informational, appraisal) proved to be an essential protective factor against vulnerability, and the effects of distress and stigmatization or discrimination [72, 80]. Most studies looked towards coworkers, management, HR, and family for support and less towards other third parties. However, access to occupational health centers and health professionals could be helpful since stigmatization and experienced distress often lead to mental health problems and negative coping mechanisms (e.g., palliative coping through alcohol or illegal substance abuse) [53, 58–60, 72, 81]. One such negative coping mechanism and also a societal factor [25] was overcompensation through hard work, which in view of well-being (at work) and together with the high emotional/psychosocial load, puts TP at risk of an imbalance between demands/rewards (Karasek model [82]) and developing burnout in the end whereby the transgender employee can still lose their job if help is not undertaken.

A recurring theme that emerged from our analysis was the preparation stage wherein trans employees educated themselves about the applicable legal protection (country legislation, company policy) and looked towards trans-friendly organizations. Our results showed that there is still much to gain at the level of legislation. UK studies [17, 35, 57] have mentioned the limitations of their equality acts and the need to develop policies beyond the existing legislation, while in the USA, the governmental structure (federal vs. state) inhibits the development of equality for all regardless of their GI, although recent executive orders may indicate future improvements [83]. While in the European union only 13 of the 31 countries have protection for GI by national legislation [84], although some large organizations (e.g., the Belgian Railways) have shown a growing awareness for an equality and diversity policy [85, 86].

As highlighted above, as perceptions in our studies were mixed so were the outcome expectations at work, which is consistent with other (LGBTQIA) literature [87, 88]. The sequence of CO and communication of the intention to TAW, which was dependent on private disclosure experiences and work relationships, was firstly done with persons considered as “safe and trustworthy”, followed by a communication through different channels. These qualitative findings are in line with Law et al. [34] wherein disclosure behaviors were predicted by, amongst others, organizational supportiveness and private CO. CO was also positively related with job satisfaction and commitment and negatively with work anxiety, but all were mediated by coworkers’ reactions (support vs. negative) [34]. In distinction with other countries, besides Belgium and the Netherlands [16], the occupational physician (OP) was rarely the first contact person, although health professionals are generally early in the disclosure sequence [89]. This could be attributed to the different role company doctors play in different countries [90–97] and partly to the general negative experiences with health care professionals [98–101], although company doctors are not specified in these reviews. This leads us to understand that although there are systems available at work for trans employees, there is an additional gap in the literature about the role of OP’s in the transition.

TAW was colored by reactions from the work environment, knowledge of transgender issues, and implementation of policies, career expectations, and the existence of a transition plan. Law et al. [34] already mentioned the importance of reactions in the work environment, especially among coworkers, as a mediator between disclosure and work outcomes. Martinez et al. [87], in their study on authentic identity expression, also placed a hierarchy onto this mediator role. The degree and support expressed by supervisors set an example for other personnel’s attitudes and may be critical in the trans employee’s well-being [87], which was also highlighted by Marvell et al. [57], wherein managers are key to an inclusive work environment. On the other hand, oppositional behavior by colleagues to improve the current work climate towards TP can also have a relational value for them and enhance work outcomes and general well-being [102]. As such, different actors of the organization and the organization can also be viewed as pivotal in the RTW process and the employee’s motivation to go back to work.

Knowledge of trans issues, as well as employment law and policies, form the basis of an inclusive work environment, and data therein were mixed. It is clear that there is a need for continuous education and training for HR personnel and managers among others to familiarize themselves with terminology and show competence and empathy towards (undisclosed) TP and, if necessary, to enlist help of legal experts. This should be accompanied by a genuine effort on the part of the organization to publicize their culture of diversity since public relations towards society is crucial to make sure that TP can comfortably apply for jobs, CO, and TAW. Furthermore, the correct implementation of diversity and antidiscrimination policies are essential, as some industries still have a (perceived) transphobic culture [16] despite having had diversity training in place. The collaboration with trans employees, derived from our results, within organizations and on a societal level could prove very fruitful on a micro, meso and macro level and is in line with the call of Beauregard et al. [103] for more transgender voices in the workplace.

Negative career outcomes or prospects were common in all of the included studies. Participants voiced their fears of dismissal, missed job opportunities and demotions, personal negative job effects (e.g., concentration), binary thinking in job expectations with the male gender as the higher position on the labor market, and cissexism or struggles on the level of infrastructure (such as dressing rooms, toilets), gender policing and discrimination by coworkers, and professional isolation. This was highlighted by Gut et al. [17] in an international survey, wherein about one quarter was demoted or fired in light of their GI, and half reported negative reactions from their work surroundings in the absence of diversity training. Although most studies in transgender (health) literature have the tendency to focus on the negative, for good reason, there were in general more positive experiences of TAW than expected, and supportive coworkers [17] and managers [17] influenced morale and a positive work culture.

From our results, the general feeling is that work adjustments and flexibility were not proficiently applied. Work adjustments are one of the core rules of RTW management after a work absence for psychosocial and medical reasons [104, 105]; therefore, it sounds very logical that an enhanced work participation through flexible adaptations can only benefit the employee and employer during TAW and diminish involuntary or unwanted work absences. Graded RTW, flexible working hours, teleworking, workplace changes, alterations in workload and job tasks, and job crafting (physical and/or task and/or cognitive) have been proficient in many psychosocial and health issues [106–114]. Job crafting refers to employees redesigning work tasks to fit their motives, passions, and strengths [112, 115, 116] which arises more frequently as employees in general experience different needs and regulatory foci at work [117], which based on our results should be added as an option to the transition plan.

### Strengths and limitations

To our knowledge, this is the first systematic review conducted on the intersectionality of RTW, transgender people, and TAW, thereby shining a light on the importance of a sustainable work resumption, the prevention of turnover, and retaining valuable productive workers. With our review, we have shown a clear gap in knowledge about RTW data, experiences, and a lack of possible job re-entry interventions.

By including diverse study methodologies, we were able to analyze a heterogeneous sample of research data, although there is still a publication bias to consider. Since we focused solely on Western studies, it is not clear if these results and the gap of knowledge concerning RTW of transgender persons are transferable to other non-Western countries.

The quality of quantitative and qualitative studies was good overall, while the mixed-methods studies were generally of a low quality (see Results section), but as stated by the MMAT recommendations [45], we did not exclude any of the studies on the basis of their quality assessment.

While most included studies involved TP in general, a select number of qualitative studies focused on either TW or TM, and in some studies specific GIs (gender fluid, nonbinary persons) were underrepresented. This can also be explained by the fact that, although included in our search string, studies solely focused on nonbinary persons but those who did not choose any steps in GAC with possible work absences or for whom there was no mention of TAW with possible work absences were discarded. Furthermore research focused on this subgroup has also just undergone a recent expansion [23, 118], and only in recent years has GI been explicitly mentioned beyond the binary when describing study populations.

We are aware of an existing selection bias and confirmation bias, as the majority of the authors of this systematic review are, apart from one sexually fluid orientation (JVdC), Caucasian, heterosexual, cisgender persons with an expertise in the occupational/vocational field. By having our manuscript co-authored by an expert (JM) in gender studies and TG health, we aimed to reduce this concern. Furthermore, throughout all studies, participants were recruited through clinics, conferences, or online. This is not uncommon in transgender research literature, as people will instinctively participate in studies by way of perceived safe spaces. The differences in definitions of transgender persons, inherent to the sociopolitical landscape, and mostly small but different sample sizes and methods, make comparisons difficult. Finally, the lack of quantitative data also prohibited us from performing a meta-analysis of RTW outcomes.

### Future research and practical implications for (occupational) health professionals and vocational experts

Future TG research would benefit from collecting information on objective work outcomes, such as sick days, career breaks, unpaid leave, and RTW patterns, through surveys and interviews at a (inter)national level. In addition, by evaluating the medical and professional history, personality, motivation, work environment characteristics, work relations, barriers and support for organizations and third parties, in particular occupational health professionals, can provide new insights for optimal guidance of trans employees in their chosen path.

Within studies involving participants identifying under the umbrella transgender term, the risk of homogenization persists, even with an emic approach. Future RTW research should refrain from only conducting studies of transgender people as one group but should focus on diverse samples prioritizing those who have been identified by prior research as higher on the intersectionality scale and who are more vulnerable. Leaning on a larger body of evidence, a more diverse and tailored RTW assistance can be achieved. In addition to the fact that more objective variables should be collected in this group, more in-depth knowledge of the experiences of transgender people is needed. Therefore, mixed and qualitative research, by means of, e.g., in-depth interviews or diary studies, is necessary to help us better understand (nonbinary) transgender people’s RTW outcomes.

A joint effort and shared responsibility of all stakeholders involved is required. Organizations should work alongside occupational health centers for assistance in policy and transition plans, while clinical professionals and counsellors should refer to their occupational colleagues who have more accurate insight into labor laws and the inner workings of the organization to which their trans client belongs. Occupational health centers, on the other hand, should provide a RTW expert and counsel equality/diversity experts, while also building on self-awareness and self-reflection.

Based on the results of this systematic review and our expertise in occupational health, we aim to create a RTW model and develop tools for health professionals to improve counselling and job retention for TP.

## Conclusion

In addition to a number of important insights, the results of this review also showed an important gap in knowledge regarding transgender issues among several stakeholders. Successful CO and TAW are likely dependent on adaptive and positive coping mechanisms, tailored genuine support from coworkers, HR and management, and the sensibilization and education of the entire workforce. External help from legal and vocational experts, better collaboration with occupational health services in developing policy and broad transition plans, including work accommodations and building mental health resilience, could achieve a more sustainable RTW and empowerment of transgender people. These findings may stimulate other researchers to include RTW in transgender health research and provide more complete occupational data from participants.

A more profound understanding of the experiences of transgender people and influencers in the intersectionality of (return) to work and transitioning through research and collaboration beyond disciplines and with the trans community is the only way forward to create a more inclusive and diverse work climate and society.

## Supporting information

S1 Checklist PRISMA checklist. (DOCX)

S1 Protocol Study protocol registered on PROSPERO. (PDF)

S1 Text Full search string PubMed. (PDF)

S1 Table Excluded articles by full-text screening. (DOCX)
