# Supplementary material for: Return to work of transgender people: A systematic review through the blender of occupational health
Source: PLoS One. 2021 Nov 1;16(11):e0259206. doi: 10.1371/journal.pone.0259206 (PMC8559954; doi:10.1371/journal.pone.0259206)
Supplement: S1 Protocol — (PDF) [file pone.0259206.s002.pdf]

National Institute for Health Research

UNIVERSITY of York
Centre for Reviews and Dissemination

# Systematic review

*Fields that have an **asterisk (*)** next to them means that they **must be answered. Word limits** are provided for each section. You will be unable to submit the form if the word limits are exceeded for any section. Registrant means the person filling out the form.*

## 1. * Review title.

Give the title of the review in English

Return to work of transgender persons: a systematic review through the blender of occupational health

## 2. Original language title.

For reviews in languages other than English, give the title in the original language. This will be displayed with the English language title.

## 3. * Anticipated or actual start date.

Give the date the systematic review started or is expected to start.

01/02/2019

## 4. * Anticipated completion date.

Give the date by which the review is expected to be completed.

01/05/2021

## 5. * Stage of review at time of this submission.

Tick the boxes to show which review tasks have been started and which have been completed. Update this field each time any amendments are made to a published record.

**Reviews that have started data extraction (at the time of initial submission) are not eligible for inclusion in PROSPERO**. If there is later evidence that incorrect status and/or completion date has been supplied, the published PROSPERO record will be marked as retracted.

This field uses answers to initial screening questions. It cannot be edited until after registration.

The review has not yet started: No

| Review stage | Started | Completed |
|---|---|---|
| Preliminary searches | Yes | Yes |
| Piloting of the study selection process | Yes | Yes |
| Formal screening of search results against eligibility criteria | Yes | Yes |
| Data extraction | Yes | Yes |
| Risk of bias (quality) assessment | Yes | Yes |
| Data analysis | Yes | Yes |

Provide any other relevant information about the stage of the review here.

Preliminary protocol written in 2019. Stage of project (april 2021) finalising manuscript

Preliminary protocol written in 2019. Stage of project (april 2021) finalising manuscript

## 6. * Named contact.

The named contact is the guarantor for the accuracy of the information in the register record. This may be any member of the review team.

Joy Van de Cauter

## Email salutation (e.g. "Dr Smith" or "Joanne") for correspondence:

Dr Van de Cauter

## 7. * Named contact email.

Give the electronic email address of the named contact.

joy.vandecauter@ugent.be

## 8. Named contact address

Give the full institutional/organisational postal address for the named contact.

Department of Public Health and Primary Care, Faculty of Medicine and Health Sciences, Ghent University

Corneel Heymanslaan 10, 4K3 (ingang 42)

9000 Ghent

Belgium

## 9. Named contact phone number.

Give the telephone number for the named contact, including international dialling code.

003293328362

## 10. * Organisational affiliation of the review.

Full title of the organisational affiliations for this review and website address if available. This field may be completed as 'None' if the review is not affiliated to any organisation.

Ghent University, Department of Public Health and Primary Care

## Organisation web address:

http://www.publichealth.ugent.be/

## 11. * Review team members and their organisational affiliations.

Give the personal details and the organisational affiliations of each member of the review team. Affiliation refers to groups or organisations to which review team members belong. **NOTE: email and country now MUST be entered for each person, unless you are amending a published record.**

Dr Joy Van de Cauter. Ghent University, Department of Public Health and Primary Care
Professor Lutgart Braeckman. Ghent University, Department of Public Health and Primary Care
Professor Dominique Van de Velde. Ghent University, Department of Rehabilitation Sciences
Hanna Van Schoorisse. Ghent University
Professor Joz Motmans. Ghent University, Department of Languages and Cultures, Faculty of Arts and Philosophy

## 12. * Funding sources/sponsors.

Details of the individuals, organizations, groups, companies or other legal entities who have funded or sponsored the review.

None

## Grant number(s)

State the funder, grant or award number and the date of award

## 13. * Conflicts of interest.

List actual or perceived conflicts of interest (financial or academic).

None

## 14. Collaborators.

Give the name and affiliation of any individuals or organisations who are working on the review but who are not listed as review team members. **NOTE: email and country must be completed for each person, unless you are amending a published record.**

## 15. * Review question.

State the review question(s) clearly and precisely. It may be appropriate to break very broad questions down into a series of related more specific questions. Questions may be framed or refined using PI(E)COS or similar where relevant.

What are the return-to-work (RTW) rates of transgender employees during and after transition?

How do transgender workers experience transitioning and RTW?

## 16. * Searches.

State the sources that will be searched (e.g. Medline). Give the search dates, and any restrictions (e.g. language or publication date). Do NOT enter the full search strategy (it may be provided as a link or attachment below.)

Relevant databases concerning health, psychological , psychiatric, sociological publications as well as grey literature will be explored systematically (including: MEDLINE, Embase, EBSCOhost, ProQuest, Scopus, and Web of Science).

Studies reporting quantitative and qualitative data of adult transgender persons combined with RTW outcomes are eligible for inclusion.

Studies published between fall of 2006, depending on the database coverage, until the March 1st 2021 were sought. Language will be restricted to English, Dutch, French and German. Hand searching of the references of key articles will be performed and references will be checked="checked" value="1" via Web of Science.

The search strategy for MEDLINE will be available in the protocol. Changes in the search strategy are database-dependent and for each database a search alert has been set up, which will be followed up. A re-run of the string will be executed before final analyses.

## 17. URL to search strategy.

Upload a file with your search strategy, or an example of a search strategy for a specific database, (including the keywords) in pdf or word format. In doing so you are consenting to the file being made publicly accessible. Or provide a URL or link to the strategy. Do NOT provide links to your search **results**.

https://www.crd.york.ac.uk/PROSPEROFILES/128395_STRATEGY_20190430.pdf

Alternatively, upload your search strategy to CRD in pdf format. Please note that by doing so you are consenting to the file being made publicly accessible.

Do not make this file publicly available until the review is complete

## 18. * Condition or domain being studied.

Give a short description of the disease, condition or healthcare domain being studied in your systematic review.

Return-to-work (RTW) : rate, time to RTW, number of sick days, RTW-experiences after social and medical transitioning (GAMI: gender affirming medical interventions ) of adult patients with gender dysphoria or whom identify as trans* (transgender persons and non-binary persons, genderfluid and genderqueer persons).

## 19. * Participants/population.

Specify the participants or populations being studied in the review. The preferred format includes details of both inclusion and exclusion criteria.

Inclusion: adults with gender dysphoria, transgender adults, transsexual adults, trans*workers/employees

Based on The Yogyakarta Principles, first published in 2006, and the subsequent developments in the political and social landscape, we decided to only include studies between 2006 and 2019.

Excusion: children, teens, informal workers, sex workers, HIV-related focus, adolescents, intersex persons, persons with dual-role transvestism

## 20. * Intervention(s), exposure(s).

Give full and clear descriptions or definitions of the interventions or the exposures to be reviewed. The preferred format includes details of both inclusion and exclusion criteria.

All gender affirming care (counselling, gender-affirming hormonal therapy and gender-affirming procedures) are considered as eligible for inclusion if information on the RTW process or experiences are available. Both conservative and invasive intervention types can be individual or combined. There will be no limits placed upon timing, frequency, dosage e.a.

## 21. * Comparator(s)/control.

Where relevant, give details of the alternatives against which the intervention/exposure will be compared (e.g. another intervention or a non-exposed control group). The preferred format includes details of both inclusion and exclusion criteria.

For quantitative data, if found, a general comparison to the RTW research domain and absenteism in European workers can be performed.

For the qualitative data a comparison is not applicable.

## 22. * Types of study to be included.

Give details of the study designs (e.g. RCT) that are eligible for inclusion in the review. The preferred format includes both inclusion and exclusion criteria. If there are no restrictions on the types of study, this should be stated.

All study designs reporting on adult transgender persons combined with RTW outcomes are eligible for inclusion. Quantitative data (RTW rate; time to RTW, sick days) will be analysed in one part of the review and qualitative data involving RTW experiences of patients will be handled separately.

## 23. Context.

Give summary details of the setting or other relevant characteristics, which help define the inclusion or exclusion criteria.

## 24. * Main outcome(s).

Give the pre-specified main (most important) outcomes of the review, including details of how the outcome is defined and measured and when these measurement are made, if these are part of the review inclusion criteria.

Return-to-work (RTW) is defined as work resumption (full-time, part-time or self-employed) which can be eligible as a dichotomous variable (yes, no), a rate/proportion, number of RTW attempts, time to RTW.

A descriptive overview of RTW will be made if possible. No limitations will be placed on the follow-up period in which RTW can occur in the selected studies.

No specific assessment tool for RTW will be upheld as an inclusion criteria.

RTW experiences of transgender persons, especially those involving facilitators and barriers to RTW, will be established based on qualitative data.

## Measures of effect

Please specify the effect measure(s) for you main outcome(s) e.g. relative risks, odds ratios, risk difference, and/or 'number needed to treat.

## 25. * Additional outcome(s).

List the pre-specified additional outcomes of the review, with a similar level of detail to that required for main outcomes. Where there are no additional outcomes please state 'None' or 'Not applicable' as appropriate to the review

Work-related outcomes such as employment, unemployment, turnover, occupational/sector characteristics, work adjustments

Age of transition , number of sick days

## Measures of effect

Please specify the effect measure(s) for you additional outcome(s) e.g. relative risks, odds ratios, risk difference, and/or 'number needed to treat.

## 26. * Data extraction (selection and coding).

Describe how studies will be selected for inclusion. State what data will be extracted or obtained. State how this will be done and recorded.

All results found through the search strategy will be downloaded in a database supplied by reference manager software (Endnote) wherein a deduplication will be performed automatically and manually.

Afterwards the references will be uploaded into Rayyan by which the selection process will be performed.

Superfluous studies, bases on title and abstract, will be independently be excluded by two reviewers.

Full text evaluation will be performed by the same two authors and disagreements will be discussed and handled by consulting a third reviewer.

Data extraction will be performed independently by two review authors in accordance to Cochrane collaboration checklist. Differences will be discussed and a third reviewer will be consulted if need be.

For each included study, at least the following will be extracted: general information, design and population, methods, participants, type of intervention (type of surgery and/or hormonal-replacement therapy), outcomes

(with measuring/reporting), results.

Cited authors will be contacted for requesting missing data if applicable.

## 27. * Risk of bias (quality) assessment.

State which characteristics of the studies will be assessed and/or any formal risk of bias/quality assessment tools that will be used.

Quality assessment will be handled through the QualSyst tool for quantitative and qualitative research. The MMAT will be used for mixed method studies. Disagreement will be resolved by consulting a third reviewer.

## 28. * Strategy for data synthesis.

Describe the methods you plan to use to synthesise data. This **must not be generic text** but should be **specific to your review** and describe how the proposed approach will be applied to your data. If meta-analysis is planned, describe the models to be used, methods to explore statistical heterogeneity, and software package to be used.

The findings of the included studies will be reported in a descriptive manner. If applicable, the overall range of RTW rate, mean time to RTW, mean number of sick days, mean number of RTW attempts will be calculated and reported.

Qualitative studies will be analysed by way of thematic synthesis (table) and reported in text.

## 29. * Analysis of subgroups or subsets.

State any planned investigation of 'subgroups'. Be clear and specific about which type of study or participant will be included in each group or covariate investigated. State the planned analytic approach.

If applicable subgroup analyses will be performed for transwomen and transmen for quantitative data.

## 30. * Type and method of review.

Select the type of review, review method and health area from the lists below.

### Type of review

Cost effectiveness
No

Diagnostic
No

Epidemiologic
No

Individual patient data (IPD) meta-analysis
No

Intervention
No

Meta-analysis
No

Methodology
No

Narrative synthesis
No

Network meta-analysis
No

Pre-clinical
No

Prevention
No

Prognostic
No

Prospective meta-analysis (PMA)
No

Review of reviews
No

Service delivery
No

Synthesis of qualitative studies
No

Systematic review
Yes

Other
No

## Health area of the review

Alcohol/substance misuse/abuse
No

Blood and immune system
No

Cancer
No

Cardiovascular
No

Care of the elderly
No

Child health
No

Complementary therapies
No

COVID-19
No

Crime and justice
No

Dental

No

Digestive system
No

Ear, nose and throat
No

Education
No

Endocrine and metabolic disorders
No

Eye disorders
No

General interest
No

Genetics
No

Health inequalities/health equity
No

Infections and infestations
No

International development
No

Mental health and behavioural conditions
No

Musculoskeletal
No

Neurological
No

Nursing
No

Obstetrics and gynaecology
No

Oral health
No

Palliative care
No

Perioperative care
No

Physiotherapy
No

Pregnancy and childbirth
No

Public health (including social determinants of health)
Yes

National Institute for Health Research

Rehabilitation
No

Respiratory disorders
No

Service delivery
No

Skin disorders
No

Social care
No

Surgery
No

Tropical Medicine
No

Urological
No

Wounds, injuries and accidents
No

Violence and abuse
No

## 31. Language.

Select each language individually to add it to the list below, use the bin icon  to remove any added in error.
English

There is an English language summary.

## 32. * Country.

Select the country in which the review is being carried out. For multi-national collaborations select all the countries involved.

Belgium

## 33. Other registration details.

Name any other organisation where the systematic review title or protocol is registered (e.g. Campbell, or The Joanna Briggs Institute) together with any unique identification number assigned by them. If extracted data will be stored and made available through a repository such as the Systematic Review Data Repository (SRDR), details and a link should be included here. If none, leave blank.

## 34. Reference and/or URL for published protocol.

If the protocol for this review is published provide details (authors, title and journal details, preferably in Vancouver format)

Add web link to the published protocol.

Or, upload your published protocol here in pdf format. Note that the upload will be publicly accessible.

No I do not make this file publicly available until the review is complete

Please note that the information required in the PROSPERO registration form must be completed in full even if access to a protocol is given.

## 35. Dissemination plans.

Do you intend to publish the review on completion?

Yes

Give brief details of plans for communicating review findings.?

## 36. Keywords.

Give words or phrases that best describe the review. Separate keywords with a semicolon or new line. Keywords help PROSPERO users find your review (keywords do not appear in the public record but are included in searches). Be as specific and precise as possible. Avoid acronyms and abbreviations unless these are in wide use.

transgender, return-to-work, (medical) transition, gender-affirming care, gender affirming medical interventions (GAMI), work experience, gender dysphoria

## 37. Details of any existing review of the same topic by the same authors.

If you are registering an update of an existing review give details of the earlier versions and include a full bibliographic reference, if available.

## 38. * Current review status.

Update review status when the review is completed and when it is published.New registrations must be ongoing so this field is not editable for initial submission.
Please provide anticipated publication date

Review_Ongoing

## 39. Any additional information.

Provide any other information relevant to the registration of this review.

To the best of our knowledge, this will be the first study to evaluate RTW of transgender people.

The findings of this systematic review will provide valuable insights into niche of knowledge around quantitative data and RTW experiences of trans employees.

This systematic review will serve as a foundation for a mixed methods project examining the barriers/facilitators of RTW in trans employees and to provide preventive tools for tailored reintegration in service of occupational physicians and enterprises.

## 40. Details of final report/publication(s) or preprints if available.

Leave empty until publication details are available OR you have a link to a preprint (NOTE: this field is not editable for initial submission). List authors, title and journal details preferably in Vancouver format.

Give the link to the published review or preprint.
