# Supplementary material for: Return to work of transgender people: A systematic review through the blender of occupational health
Source: PLoS One. 2021 Nov 1;16(11):e0259206. doi: 10.1371/journal.pone.0259206 (PMC8559954; doi:10.1371/journal.pone.0259206)
Supplement: S1 Text — (PDF) [file pone.0259206.s003.pdf]

S3 Text. Full electronic search string for Pubmed interface

((((((((((((("Sex Reassignment Procedures"[Mesh] OR "Sex Reassignment Surgery"[Mesh]) OR "sex reassignment"[Title/Abstract]) OR ("sex change"[Title/Abstract] OR "sex changed"[Title/Abstract] OR "sex changer"[Title/Abstract] OR "sex changers"[Title/Abstract] OR "sex changes"[Title/Abstract])) OR "sex change procedure"[Title/Abstract]) OR "confirmation surgery"[Title/Abstract]) OR ("gender change"[Title/Abstract] OR "gender changes"[Title/Abstract])) OR "gender confirmation"[Title/Abstract]) OR "gender reassignment"[Title/Abstract]) OR ("sex transformation"[Title/Abstract] OR "sex transformations"[Title/Abstract])) OR "sex alteration"[Title/Abstract]) OR ((("transgender persons"[MeSH Terms] OR ("transgender"[Title/Abstract] AND "persons"[Title/Abstract]) OR "transgender persons"[Title/Abstract] OR "transgender"[Title/Abstract]) OR ("transsexualism"[MeSH Terms] OR "transsexualism"[Title/Abstract] OR "transgenderism"[Title/Abstract])) OR ((((((((((((("transsex"[Title/Abstract] OR "transsexed"[Title/Abstract] OR "transsexism"[Title/Abstract] OR "transsexual"[Title/Abstract] OR "transsexual'"[Title/Abstract] OR "transsexual's"[Title/Abstract] OR "transsexualiam"[Title/Abstract] OR "transsexualism"[Title/Abstract] OR "transsexualism'"[Title/Abstract] OR "transsexualism's"[Title/Abstract] OR "transsexualisme"[Title/Abstract] OR "transsexualisms"[Title/Abstract] OR "transsexualists"[Title/Abstract] OR "transsexuality"[Title/Abstract] OR "transsexualization"[Title/Abstract] OR "transsexually"[Title/Abstract] OR "transsexuals"[Title/Abstract] OR "transsexuals'"[Title/Abstract] OR "transsexuel"[Title/Abstract] OR "transsexuell"[Title/Abstract] OR "transsexuellengesetz"[Title/Abstract] OR "transsexulaism"[Title/Abstract] OR "transsexulism"[Title/Abstract]) OR ("transgender"[Title/Abstract] OR "transgender'"[Title/Abstract] OR "transgender's"[Title/Abstract] OR "transgendered"[Title/Abstract] OR "transgenderism"[Title/Abstract] OR "transgenderists"[Title/Abstract] OR "transgenders"[Title/Abstract])) OR "transpersons"[Title/Abstract]) OR ("gender variant"[Title/Abstract] OR "gender variants"[Title/Abstract])) OR ("gender queer"[Title/Abstract] OR "gender queering"[Title/Abstract])) OR ("male to female"[Title/Abstract] OR "male to females"[Title/Abstract])) OR ("transwomen"[Title/Abstract] OR "transwomen's"[Title/Abstract]) OR ("transmen"[Title/abstract] OR "transmen's"[Title/Abstract]) OR ("gender non conforming"[Title/Abstract] OR "gender non conformity"[Title/Abstract])) OR ("two spirit"[Title/Abstract] OR "two spirited"[Title/Abstract] OR "two spirits"[Title/Abstract])) OR "gender diverse"[Title/Abstract]) OR ("gender fluid"[Title/Abstract] OR "gender fluidity"[Title/Abstract])) OR "non binary"[Title/Abstract]) OR ("female to male"[Title/Abstract] OR "female to males"[Title/Abstract])) OR "gender variant persons"[Title/Abstract] OR "lgbt"[All Fields])) OR (("gender dysphoria"[MeSH Terms] OR ("gender"[Title/Abstract] AND "dysphoria"[Title/Abstract]) OR "gender dysphoria"[Title/Abstract]) OR (((("gender dysphoric"[Text Word] OR "gender incongruence"[Text Word]) OR "gender identity disorder"[Text Word]) OR "gender-variant"[Text Word]) OR "gender transition"[Text Word]))) OR ((cross-sex[Title/Abstract] AND ("hormones"[Pharmacological Action] OR "hormones"[MeSH Terms] OR "hormones"[Title/Abstract] OR "hormone"[Title/Abstract]) AND ("therapy"[Subheading] OR "therapy"[Title/Abstract] OR "treatment"[Title/Abstract] OR "therapeutics"[MeSH Terms] OR "therapeutics"[Title/Abstract])) OR (("sex"[MeSH Terms] OR "sex"[Title/Abstract] OR "gender"[Title/Abstract] OR "gender identity"[MeSH Terms] OR ("gender"[Title/Abstract] AND "identity"[Title/Abstract]) OR "gender identity"[Title/Abstract]) AND affirming[Title/Abstract] AND ("hormones"[Pharmacological Action] OR "hormones"[MeSH Terms] OR "hormones"[Title/Abstract] OR "hormone"[Title/Abstract]) AND ("therapy"[Subheading] OR "therapy"[Title/Abstract] OR "therapeutics"[MeSH Terms] OR "therapeutics"[Title/Abstract]))) OR ("cross sex hormone

therapy"[Text Word] OR "cross sex hormone treatment"[Text Word]) OR "cross sex hormone use"[Text Word] OR "cross sex identity"[Text Word] OR "gender affirming hormones"[Text Word] OR "gender affirming hormone therapy"[Text Word]) AND (((((((("return to work"[Text Word] OR "return to work"[MeSH Terms]) OR "work"[Text Word]) OR "work"[MeSH Terms]) OR "employment"[MeSH Terms]) OR "employment"[Text Word]) OR "employed"[Text Word]) OR "employee"[Text Word]) OR "work retention"[Text Word]) OR "work resumption"[Text Word]) OR "back to work"[Text Word])
