# Supplementary material for: Return to work of transgender people: A systematic review through the blender of occupational health
Source: PLoS One. 2021 Nov 1;16(11):e0259206. doi: 10.1371/journal.pone.0259206 (PMC8559954; doi:10.1371/journal.pone.0259206)
Supplement: S1 Table — (DOCX) [file pone.0259206.s004.docx]

**S4 Table Excluded articles by full text screening**

| **N°** | **Reference** | **Summary comment for exclusion** | **Type of exclusion** |
| --- | --- | --- | --- |
| **1** | Ahmad S, Joseph J, Kim S, et al. A cross-sectional study of clinical and psychosocial characteristics of a cohort of 371 transgender patient treated at Albany medical center. Endocrine Reviews 2016; 37. | No full text article, only poster available, no focus on RTW or sick leave information | Unavailable  Wrong outcome |
| **2** | Amaral AS, Covelo V, Marques-Pinto A, et al. Stigma perception and controversies among gender dysphoric individuals in a Portuguese outpatient unit. 19th Congress of the European Society for Sexual Medicine, ESSM 2017. Nice: J. Sex. Med, 2017, p. e146. | No RTW, focus on stigma and discrimination | Wrong  outcome |
| **3** | Andréasson M, Georgas K, El, et al. Patient-reported outcome measures used in gender confirmation surgery: a systematic review. Plastic and reconstructive surgery 2018; 141: 1026-1039. | No work focus | Wrong outcome |
| **4** | Baker SJ and Lucas K. Is it safe to bring myself to work? Understanding LGBTQ experiences of workplace dignity. Canadian Journal of Administrative Sciences-Revue Canadienne Des Sciences De L Administration 2017; 34: 133-148. | More about discrimination and honor | Wrong outcome |
| **5** | Bandecchi C, Deiana V, Pinna F, et al. Socio-demographic features of gender dysphoria in a Sardinian adult population. European Psychiatry 2016; 33: S588. | No RTW | Wrong outcome |
| **6** | Barnard S and Dainty A. Coming out and staying in: how sexual orientation and gender identity matters in construction employment. Proceedings of the Institution of Civil Engineers-Municipal Engineer 2018; 171: 141-148. | More general LGBT results, few separate results of trans people, more focus on general CO, no RTW or sick leave information | Wrong population  Wrong outcome |
| **7** | Barone M, Cogli, ro A, et al. A Systematic review of patient-reported outcome measures following transsexual surgery. Aesthetic plastic surgery 2017; 41: 700-713. | No work focus or sick leave information | Wrong outcome |
| **8** | Beagan BL, De Souza L, Godbout C, et al. “This is the biggest thing you'll ever do in your life”: Exploring the occupations of transgendered people. Journal of Occupational Science 2012; 19: 226-240. | No RTW | Wrong outcome |
| **9** | Bertrand B, Perchenet AS, Colson TR, et al. Female-to-male transgender chest reconstruction: A retrospective study of patient satisfaction. Ann Chir Plast Esthet 2017; 62: 303-307. | No RTW, no work experiences or sick leave information | Wrong outcome |
| **10** | Bouman MB, van Zeijl MC, Buncamper ME, et al. Intestinal vaginoplasty revisited: a review of surgical techniques, complications, and sexual function. The journal of sexual medicine 2014; 11: 1835-1847. | Focus surgically methods | Wrong outcome |
| **11** | Chech A. F., Rothwell R. W. LGBT workplace inequality in the federal workforce: intersectional processes, organizational contexts, and turnover considerations. ILR Review 2020; 73(1): 25-60. | No LGBT stratification, general LGBT results of work place inequalities | Wrong population  Wrong outcome |
| **12** | Cheung AS, Ooi O, Davidoff D, et al. Clinical characteristics of trans and gender diverse individuals attending specialist endocrinology clinics. Endocrine Society of Australia Annual Scientific Meeting 2017. Perth, WA, Australia: Clinical Endocrinology. 2018, p. 57-58. | Conference abstract, No work focus, no sick leave information | Wrong outcome |
| **13** | Christian R and Lee R. The Colorado transgender health survey. Journal of General Internal Medicine 2015; 30: S267-S268. | No RTW or sick leave information | Wrong outcome |
| **14** | Chu J, Havil, M, et al. Barriers to care and reproductive considerations for female-to-male transgender gender confirmation surgery. 44th Annual meeting of the Society Of Gynecologic Surgeons, SGS 2018. Orlando, FL, United States: American Journal of Obstetrics and Gynecology, 2018, p. S915-S916. | Focus on surgery, no sick leave information, no RTW | Wrong outcome |
| **15** | Collins JC, McFadden C, Rocco TS, et al. The problem of transgender marginalization and exclusion: critical actions for human resource development. Human Resource Development Review 2015; 14: 205-226. | Theoretical paper focused on HR (critical thinking) | Wrong outcome |
| **16** | Coll-Planas G and Misse M. Identification of the factors influencing the job integration of transgender people. Exploring the case of Barcelona. Obets-Revista De Ciencias Sociales 2018; 13: 45-68. | Predominantly in Spanish, No RTW focus, more focus on factors of vulnerability within society, social integration and discrimination of hiring procedures | Wrong outcome  Wrong language |
| **17** | Davidson S. Gender inequality: Nonbinary transgender people in the workplace. Cogent Social Sciences 2016; 2. | Too general, based on the National Transgender Discrimination Survey 2011, results of Transgender people and non-binary persons, more focus on discrimination and job outcome or hiring procedures, no RTW, no focus on transition at work, no (sick) leave | Wrong outcome |
| **18** | dickey LM, Walinsky D, Rofkahr C, et al. Career decision self-efficacy of transgender people: pre- and post-transition. Career Development Quarterly 2016; 64: 360-372. | More about career guidance | Wrong outcome |
| **19** | Dietert M and Dentice D. Gender identity issues and workplace discrimination: the transgender experience. Journal of Workplace Rights 2009; 14: 121-140. | Too general, focus on discrimination | Wrong outcome |
| **20** | Dispenza F, Watson LB, Chung YB, et al. Experience of career-related discrimination for female-to-male transgender persons: A Qualitative Study. Career Development Quarterly 2012; 60: 65-81. | No RTW, study focus solely on discrimination in all forms and its impact on career development | Wrong outcome |
| **21** | Djordjevic ML and Bizic MR. Sexual reassignment surgery: male to female. School of Medicine, University of Belgrade, Serbia: Nova Science Publishers, Inc., 2014, p.109-126. | Focus surgery, outcomes, no RTW, no sick leave information | Wrong outcome |
| **22** | Dreher PC, Edwards D, Hager S, et al. Complications of the neovagina in male-to-female transgender surgery: a systematic review and meta-analysis with discussion of management. Clinical anatomy (New York, NY) 2018; 31: 191-199. | Focus surgery outcomes, no RTW, no sick leave information | Wrong outcome |
| **23** | Drydakis N. Transgenderism, sex reassignment surgery and employees’ job-satisfaction. Department of Economics and International Business, Lord Ashcroft International Business School, Anglia Ruskin University, Cambridge, United Kingdom Institute for the Study of Labor, IZA, Forschungsinstitut zur Zukunft der Arbeit GmbH, Bonn, Germany: Springer International Publishing, 2016, p.83-99. | No RTW data or experiences | Wrong outcome |
| **24** | Duisin D, Nikolic-Balkoski G and Batinic B. Sociodemographic profile of transsexual patients. Psychiatr Danub 2009; 21: 220-223. | Only profile, no RTW or work experiences | Wrong outcome |
| **25** | Fabbre VD. Gender transitions in later life: the significance of time in queer aging. Journal of gerontological social work 2014; 57: 161-175. | Focus gerontology, no work experiences, no RTW | Wrong outcome |
| **26** | Fiore-Urizar M, Patel N, Bettadahalli S, et al. Demographic, clinical, and psychiatric characteristics of transsexual patients: Possible influence of age at treatment on mood and adjustment disorders. Endocrine Reviews 2011; 32. | No full text article, focus on psychiatry, no RTW or sick leave information | Unavailable  Wrong outcome |
| **27** | Forshee AS. Transgender men: A demographic snapshot. Journal of Gay and Lesbian Social Services 2008; 20: 221-236. | Only demographic profile and overview of chosen steps in GAC, no RTW, no sick leave information, | Wrong outcome |
| **28** | Frey JD, Poudrier G, Chiodo MV, et al. A systematic review of metoidioplasty and radial forearm flap phalloplasty in female-to-male transgender genital reconstruction: Is the "ideal" neophallus an achievable goal? Plastic and reconstructive surgery Global open 2016; 4: e1131. | No RTW focus, no sick leave information, focus on surgery outcomes | Wrong outcome |
| **29** | Gacilo J, Steinheider B, Stone TH, et al. The double-edged sword of having a unique perspective: Feelings of discrimination and perceived career advantages among LGBT employees. Equality, Diversity & Inclusion 2018; 37: 298-312. | No RTW focus, no stratification, more focus on perspectives and possible influence on work when taken into account by employer | Wrong population  Wrong outcome |
| **30** | Gallarda T, Rari E, Coussinoux S, et al. Gender identity disorders: Personalized care for patients seeking sex reassignment surgery. European Archives of Psychiatry and Clinical Neuroscience 2010; 260: S74. | Abstract, No RTW or work data or experiences, more focus on history of gender dysphoria and diagnosis | Wrong outcome  Unavailable |
| **31** | Garcia M. Building an academic medical center multidisciplinary genital gender affirming surgery program: The University of California San Francisco experience-initial outcomes and lessons learned. 22nd Annual Fall Scientific Meeting of the Sexual Medicine Society of North America, SMSNA 2016. Scottsdale, AZ, United States: Journal of Sexual Medicine, 2017, p. e26. | Abstract only, no RTW, focus on surgical outcomes, health insurance coverage experiences | Wrong outcome |
| **32** | Georgas K, Belgrano V, Andreasson M, et al. Bowel vaginoplasty: a systematic review. Journal of plastic surgery and hand surgery 2018: 1-9. | Focus surgery, no RTW or sick leave information or work experiences | Wrong outcome |
| **33** | Giami A and Beaubatie E. Gender identification and sex reassignment surgery in the trans population: a survey study in France. Arch Sex Behav 2014; 43: 1491-1501. | No RTW or work focus | Wrong outcome |
| **34** | Goddard JC, Vickery RM and Terry TR. Development of feminizing genitoplasty for gender dysphoria. J Sex Med 2007; 4: 981-989. | Focus plastic surgery | Wrong outcome |
| **35** | Gomez-Gil E, Trilla A, Salamero M, et al. Sociodemographic, clinical, and psychiatric characteristics of transsexuals from Spain. Arch Sex Behav 2009; 38: 378-392. | Only profile, no RTW, no sick leave information | Wrong outcome |
| **36** | Gomez-Gil E, Zubiaurre-Elorza L, de Antonio IE, et al. Determinants of quality of life in Spanish transsexuals attending a gender unit before genital sex reassignment surgery. Qual Life Res 2014; 23: 669-676. | No RTW, no sick days information | Wrong outcome |
| **37** | Guzman-Parra J, Sanchez-Alvarez N, de Diego-Otero Y, et al. Sociodemographic Characteristics and Psychological Adjustment Among Transsexuals in Spain. Archives of Sexual Behavior 2016; 45: 587-596. | Only profile, no RTW | Wrong outcome |
| **38** | Harley DA and Teaster PB. LGBT intersection of age and sexual identity in the workplace. University of Kentucky, Lexington, United States Virginia Tech, Blacksburg, VA, United States: Springer International Publishing, 2016, p.551-562. | No (return to)work focus, no LGBT stratification | Wrong population  Wrong outcome |
| **39** | Herman JL, Wilson BD and Becker T. Demographic and Health Characteristics of Transgender Adults in California: Findings from the 2015-2016 California Health Interview Survey. 2017 2017. | No (return to) work focus, no sick leave information | Wrong outcome |
| **40** | Horbach SE, Bouman MB, Smit JM, et al. Outcome of vaginoplasty in male-to-female transgenders: a systematic review of surgical techniques. The journal of sexual medicine 2015; 12: 1499-1512. | Focus surgical techniques, no (return to) work data or sick leave information | Wrong outcome |
| **41** | Hur H. The role of inclusive work environment practices in promoting LGBT employee job satisfaction and commitment. Public money & management 2020;40 (6): 423-436 | No LGBT stratification, no RTW, focus on social exchange theory related to effect of practices on job satisfaction and commitment | Wrong population  Wrong outcome |
| **42** | Karpel L, Gardel B, Revol M, et al. Psychological and sexual well being of 207 transsexuals after sex reassignment in France. Annales Medico-Psychologiques 2015; 173: 511-519. | No (return to) work or sick leave information or experiences | Wrong outcome |
| **43** | Kreukels BP, Haraldsen IR, De Cuypere G, et al. A European network for the investigation of gender incongruence: the ENIGI initiative. Eur Psychiatry 2012; 27: 445-450. | No RTW, no sick days information, focus on diagnostic procedures | Wrong outcome |
| **44** | Lehtonen J. Experiences of non-heterosexual and trans youth on career choice and in the workplace. In: T. Köllen, editor. Sexual orientation and transgender issues in organizations.Switzerland: Springer International Publishing; 2016. pp.289-306. | Focus on youth (non-heterosexual and trans), career choices | Wrong population  Wrong outcome |
| **45** | Manrique OJ, Adabi K, Martinez-Jorge J, et al. Complications and patient-reported outcomes in male-to-female vaginoplasty-where we are today: a systematic review and meta-analysis. Annals of plastic surgery 2018; 80: 684-691. | No RTW, focus on surgery outcomes, no sick leave information | Wrong outcome |
| **46** | Markovic Zigic D, Maksimovic K, Borovnica V, et al. Inpatient care of transsexual patients: Recent experience. European Psychiatry 2015; 30: 1774. | No RTW, focus on comorbid and concomitant psychiatric disorders and follow-up through case history review | Wrong outcome |
| **47** | Martinez LR, Sawyer KB and Wilson MC. Understanding the experiences, attitudes, and behaviors of sexual orientation and gender identity minority employees. Journal of Vocational Behavior 2017; 103: 1-6. | Editorial with overview of general LGBTQIA population, focus on management and organizational psychology, No (return to) work focus, no sick days information | Wrong population  Wrong outcome |
| **48** | Massino J. Workers under construction: Gender, identity, and women’s experiences of work in state socialist Romania. History Department, Northwestern University, United States: Palgrave Macmillan, 2009, p.13-31. | No trans people, focus on women and feminism | Wrong population  Wrong outcome |
| **49** | McFadden C and Crowley-Henry M. 'My People': the potential of LGBT employee networks in reducing stigmatization and providing voice. International Journal of Human Resource Management 2018; 29: 1056-1081. | Review with general LGBT results and focus on stigma | Wrong population  Wrong outcome |
| **50** | McFadden C. Lesbian, gay, bisexual, and transgender careers and human resource development: a systematic literature review. Human Resource Development Review 2015; 14: 125-162. | Review with general LGBT results, more focus on management, no RTW, no new studies or data to be used | Wrong population  Wrong outcome |
| **51** | Melsens E, Willaert W, Van Nieuwenhove Y, et al. Vaginal elongation with descending colon in patients with gender identity disorder. European Surgical Research 2014; 52: 114-115. | No RTW, more focus in surgery outcomes | Wrong outcome |
| **52** | Mizock L and Mueser KT. Employment, mental health, internalized stigma, and coping with transphobia among transgender individuals. Psychology of Sexual Orientation and Gender Diversity 2014; 1: 146-158. | No RTW, more focus on stigma | Wrong outcome |
| **53** | Morrison SD, Shakir A, Vyas KS, et al. Phalloplasty: A review of techniques and outcomes. Plastic and reconstructive surgery 2016; 138: 594-615. | Focus surgery techniques and outcomes, No RTW, no sick leave information | Wrong outcome |
| **54** | Morrison SD, Vyas KS, Motakef S, et al. Facial feminization: systematic review of the literature. Plastic and reconstructive surgery 2016; 137: 1759-1770. | Review with focus on surgery | Wrong outcome |
| **55** | Motmans J, Meier P, Ponnet K, et al. Female and male transgender quality of life: Socioeconomic and medical differences. Journal of Sexual Medicine 2012; 9: 743-750. | No RTW | Wrong outcome |
| **56** | Motmans J, Ponnet K and De Cuypere G. Sociodemographic characteristics of trans persons in Belgium: a secondary data analysis of medical, state, and social data. Arch Sex Behav 2015; 44: 1289-1299. | No RTW | Wrong outcome |
| **57** | Murad MH, Elamin MB, Garcia MZ, et al. Hormonal therapy and sex reassignment: a systematic review and meta-analysis of quality of life and psychosocial outcomes. Clinical Endocrinology 2010; 72: 214-231. | Review without work-related studies, no RTW, more focus on psychological symptoms and sexual function | Wrong outcome |
| **58** | Pell C, Prone I and Vlahakis E. A clinical audit of male to female (MTF) transgender patients attending taylor square private clinic in Sydney, Australia, aiming to improve quality of care. Journal of Sexual Medicine 2011; 8: 179. | No full text article, only conference abstract | Unavailable |
| **59** | Perovic S and Djinovic R. Genitoplasty in male-to-female transsexuals. Curr Opin Urol 2009; 19: 571-576. | No RTW, no work focus or experiences, no sick leave information, focus on surgical outcomes and techniques | Wrong outcome |
| **60** | Phoenix N and Ghul R. Gender transition in the workplace: an occupational therapy perspective. Work 2016; 55: 197-205. | Narrative review of literature between 2002-2013, no new primary articles or data to use (versus already included studies), not original study, part of data collection before Yogyakarta principles | Wrong time frame  Not original study |
| **61** | Pichler S and Ruggs EN. LGBT workers. In A. J. Colella & E. B. King , editors.The Oxford handbook of workplace discrimination. United States: Oxford University Press, 2018, p.177-196. | Narrative review with focus on focus on discrimination of general LGBT population, results, no RTW | Wrong population  Wrong outcome |
| **62** | Quick L, Morris R, Morris M, et al. Outcomes of gender dysphoria treatment for trans men and trans women in the UK national health service. Journal of Sexual Medicine 2015; 12: 14-15. | Focus on surgery outcomes & general health outcomes, No RTW, , no sick leave information | Wrong outcome |
| **63** | Reed HM, Yanes RE, Delto JC, et al. Non-grafted vaginal depth augmentation for transgender atresia, our experience and survey of related procedures. Aesthetic Plast Surg 2015; 39: 733-744. | Focus plastic surgery techniques | Wrong outcome |
| **64** | Remington AC, Morrison SD, Massie JP, et al. Outcomes After Phalloplasty: Do transgender patients and multiple urethral procedures carry a higher rate of complication? Plastic and reconstructive surgery 2018; 141: 220e-229e. | Focus surgery outcomes | Wrong outcome |
| **65** | Sawyer K, Thoroughgood C and Webster J. Queering the gender binary: understanding transgender workplace experiences. In: T. Köllen, editor. Sexual orientation and transgender issues in organizations. Switzerland: Springer International Publishing; 2016. pp.21-42. | Narrative review focused on law and discrimination and best practices and future research, no new studies from this review | Wrong outcome |
| **66** | Schilt K and Connell C. Do workplace gender transitions make gender trouble.? Gender Work and Organization 2007; 14: 596-618. | Sociology, more focus of effect of gender transitions on co-workers, binary expectations, social interactions, time period data collection 2003-2005, before Yogyakarta principles | Wrong outcome  Wrong time frame |
| **67** | Sehnal B, Sottner O, Zahumensky J, et al. Comparison of three hysterectomy methods in a set of female to male transsexuals. Geburtshilfe und Frauenheilkunde 2008; 68: 625-628. | Not work-related, focus on surgical techniques, no RTW or sick leave information | Wrong outcome |
| **68** | Sicca LM, Bizjak D and Fruttaldo A. Book review : LGBTIQ+ perspectives in institutional contexts: challenging heteronormative paradigms in the workplace. LGBT+ Perspectives. Report no. 09686673, 2017 2017. | Book review with more philosophical content, no work outcomes and no RTW, general LGBTQIA, no separate results of transgender people | Wrong population  Wrong outcome |
| **69** | Stewart L, O'Halloran P and Oates J. Investigating the social integration and wellbeing of transgender individuals: A meta-synthesis. International Journal of Transgenderism 2018; 19: 46-58. | No RTW or work focus, focus on discrimination & stigma, self-isolation, increased risk on substance abuse, homelessness &prostitution | Wrong outcome |
| **70** | Sutcliffe PA, Dixon S, Akehurst RL, et al. Evaluation of surgical procedures for sex reassignment: a systematic review. Journal of plastic, reconstructive & aesthetic surgery : JPRAS 2009; 62: 294-306; discussion 306-298. | Focus surgically methods, no RTW experiences or RTW data | Wrong outcome |
| **71** | Takács J. LGBT employees in the Hungarian labor market. Institute of Sociology, Centre for Social Sciences, Hungarian Academy of Sciences, Budapest, Hungary: Springer International Publishing, 2016, p.233-252. | No RTW focus, no stratification of transgender people | Wrong population  Wrong outcome |
| **72** | van de Grift T. Transgender individuals, medical transition and the influence on work. Tijdschrift voor Bedrijfs- en Verzekeringsgeneeskunde 2018; 26: 222-227. | Systematic review with more focus on medical interventions within GAC, No RTW or experiences, no sick leave or work absence | Wrong outcome |
| **73** | Van Schuylenbergh J and Motmans J. Transgenders and work in Belgium: Definitions, concepts and figures. Tijdschrift voor Bedrijfs- en Verzekeringsgeneeskunde 2018; 26: 218-221. | More focus on the concept of gender identity, more focus on legislative information, access to GAC, financial deprivation, unemployment and work discrimination, inclusion of cross dressers in sample population, general data for the whole group | Wrong population  Wrong outcome |
| **74** | Waite S., Ecker J., Ross E. L.. A systematic review and thematic synthesis of Canada’s LGBTQ2S+ employment, labour market and earnings literature. PLoS ONE 2019;14(10); 1-20. | General LGBTQ2S data, focus on outness and sexual orientation, wrong focus, one study of TW teachers in wrong time period (before Yogyakarta principles) and focused on CO | Wrong timeframe  Wrong outcome  Wrong population |
| **75** | Walinsky D and Whitcomb D. Using the ACA competencies for counseling with transgender clients to increase rural transgender well-being. Journal of LGBT Issues in Counseling 2010; 4: 160-175. | No transgender persons included, about counselors | Wrong population |
| **76** | Webster JR, Adams GA, Maranto CL, et al. Workplace contextual supports for LGBT employees: A review, meta‐analysis, and agenda for future research. Human Resource Management 2018; 57: 193-210. | Systematic review without new articles to include because of no RTW, no work experience or work outcome, more on attitudes and disclosure and discrimination, few separate results of transgender people (3 of which 1 study was already included and 2 studies focused on discrimination) | Wrong population  Wrong outcome |
| **77** | Wolter A, Diedrichson J, Scholz T, et al. Sexual reassignment surgery in female-to-male transsexuals: an algorithm for subcutaneous mastectomy. J Plast Reconstr Aesthet Surg 2015; 68: 184-191. | Focus on surgery outcomes | Wrong focus |

RTW : return to work

CO: coming out

GAC: gender affirming care

GAS: gender affirming surgery
